# Supplementary figures and images for: Longitudinal monitoring of KRAS-mutated circulating tumor DNA enables the prediction of prognosis and therapeutic responses in patients with pancreatic cancer
Source: PLoS One. 2019 Dec 31;14(12):e0227366. doi: 10.1371/journal.pone.0227366 (PMC6938323; doi:10.1371/journal.pone.0227366)

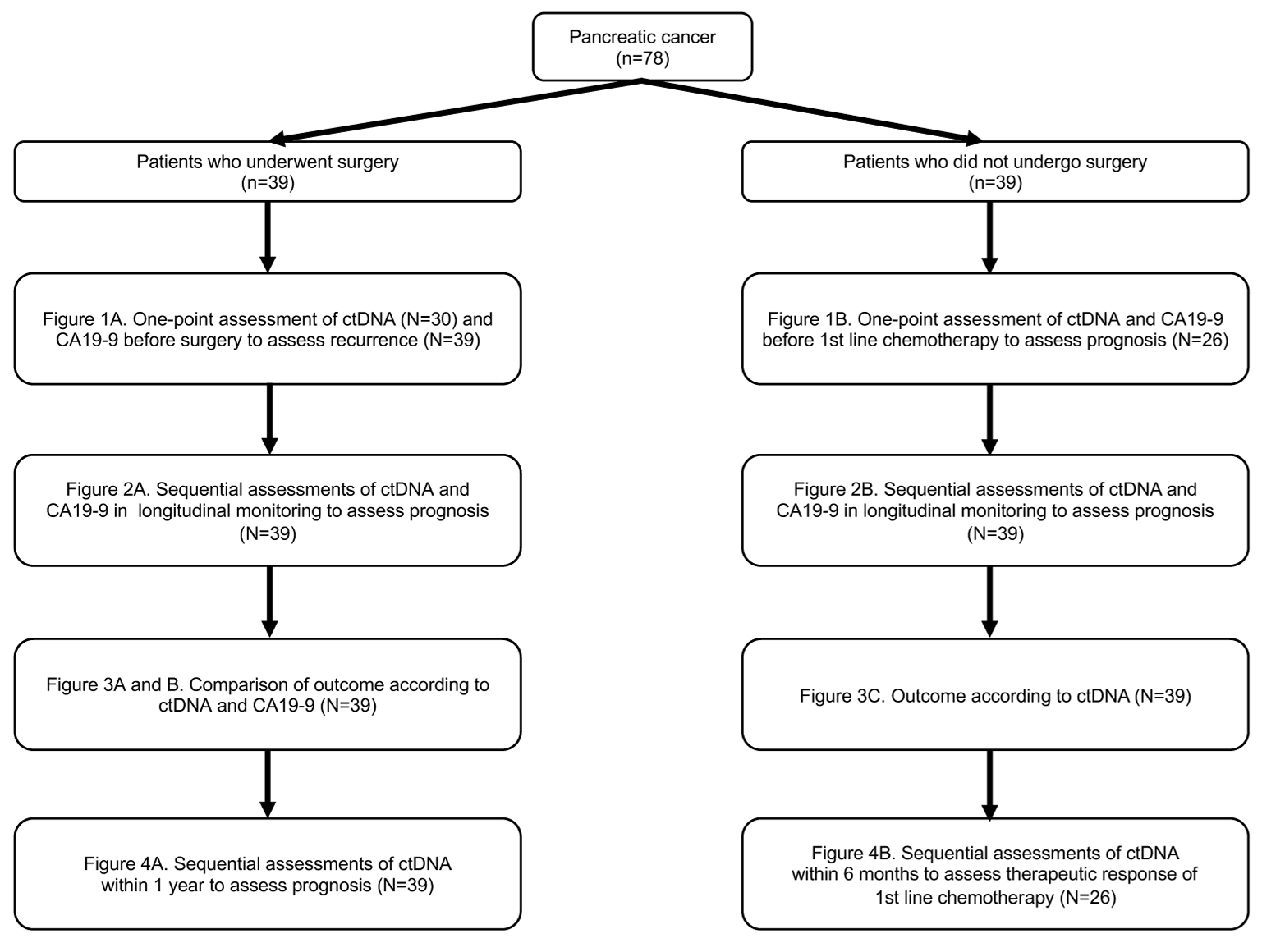

Supplement: S1 Fig — (TIF) [file pone.0227366.s001.tif]

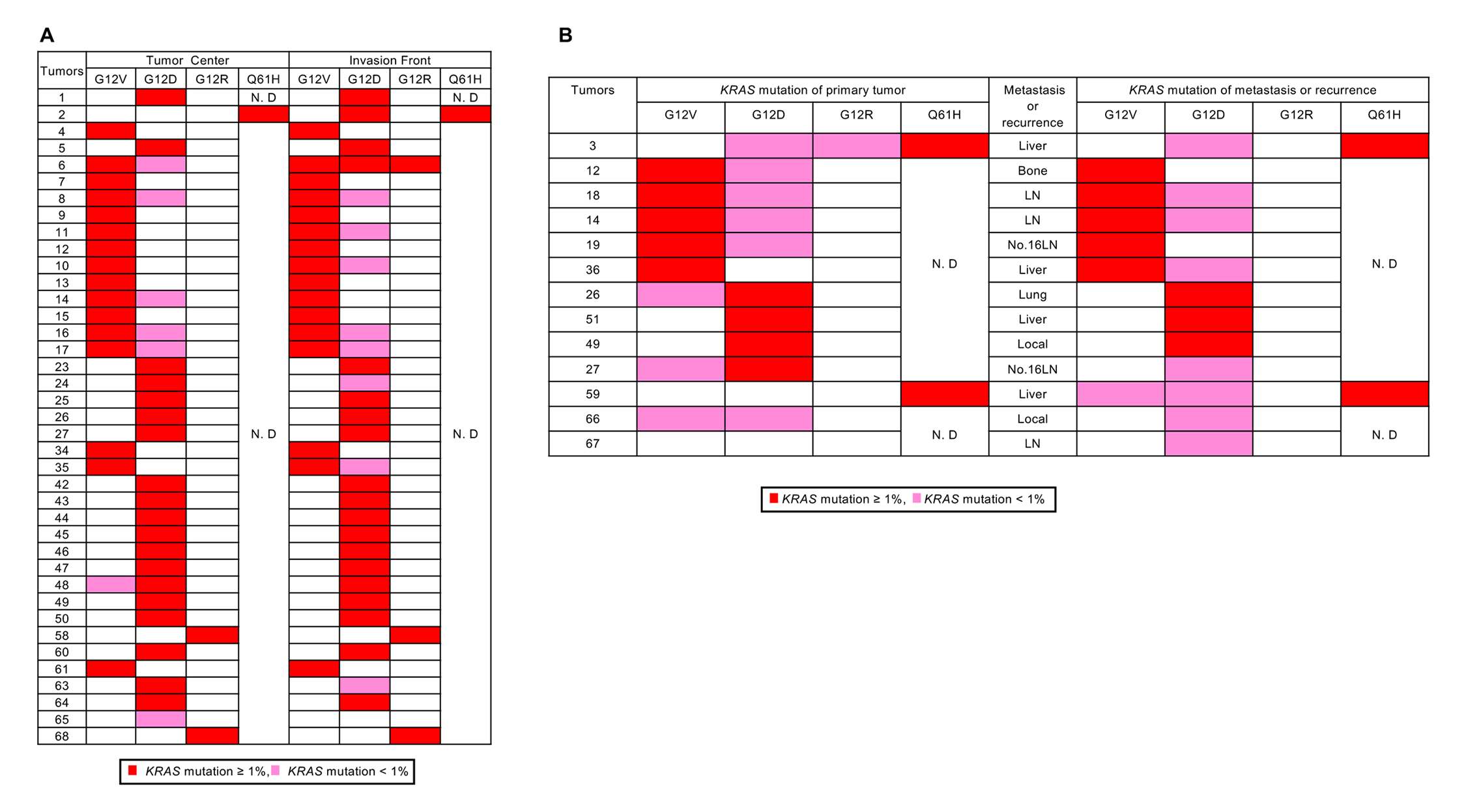

Supplement: S4 Fig — (A) Assessment of KRAS mutations between tumor center and invasion front using droplet digital polymerase chain reaction (ddPCR). KRAS mutations with frequencies ≥1% are indicated in red, whereas those with frequencies <1% are displayed in pink. Blank indicates no detection of KRAS mutation. ND, not determined. As for KRAS mutations with frequencies ≥1%, 36 tumors showed concordance between the tumor center and invasion front, accounting for 94.7%. Two tumors (no. 24 and no. 63) did not show concordance; the number of tumors corresponded to that presented in S3 Fig (B) KRAS mutations with frequencies ≥1% are indicated in red, whereas those with frequencies <1% are displayed in pink. Blank indicates no detection of KRAS mutation. ND, not determined; LN, lymph node; Local, local recurrence in residual pancreas. As for KRAS mutations with frequencies ≥1%, 10 tumors showed concordance between the primary tumor and metastasis, accounting for 90.9%. One tumor (no. 27) did not show concordance; the number of tumors corresponded to that presented in S3 Fig. (TIF) [file pone.0227366.s004.tif]
