# Supplementary material for: Longitudinal monitoring of KRAS-mutated circulating tumor DNA enables the prediction of prognosis and therapeutic responses in patients with pancreatic cancer
Source: PLoS One. 2019 Dec 31;14(12):e0227366. doi: 10.1371/journal.pone.0227366 (PMC6938323; doi:10.1371/journal.pone.0227366)
Supplement: S1 Table — A) Characteristics of patients who underwent surgery. B) Characteristics of patients who did not undergo surgery. (DOC) [file pone.0227366.s006.doc]

**Supplementary Table S1a.** Characteristics of patients who underwent surgery

| Characteristics | Value |
| --- | --- |
|  | (N=39) |
| Sex |  |
| Male | 21 (53.8%) |
| Female | 18 (46.2%) |
| Age at surgery (median, 69.5 years) | |
| >70 years | 19 (48.7%) |
| ≤70 years | 20 (51.3%) |
| Neoadjuvant chemotherapy |  |
| Yes | 7 (17.9%) |
| No | 32 (82.1%) |
| Operation methods |  |
| SSPPD and total pancreatectomy | 23 (59.0%) |
| Distal pancreatectomy | 16 (41.0%) |
| Tumor size |  |
| ≥2 cm | 10 (25.6%) |
| <2 cm | 29 (74.6%) |
| Pathological differentiation |  |
| tub1 and pap | 26 (66.7%) |
| tub2 and others^a^ | 13 (33.3%) |
| AJCC T factor |  |
| T1/T2 | 6 (15.4%) |
| T3/T4 | 33 (84.6%) |
| Lymph node metastasis |  |
| Positive | 25 (64.1%) |
| Negative | 14 (35.9%) |
| AJCC stage |  |
| IA/IB/IIA | 14 (35.9%) |
| IIB/III/IV | 25 (64.1%) |
| Preoperative CA19-9 level |  |
| ≥37 U/mL | 11 (28.2%) |
| <37 U/mL | 28 (71.8%) |
| Recurrence |  |
| Yes | 22 (56.4%) |
| No | 17 (43.6%) |
| Chemotherapy after recurrence | |
| Yes | 18 (46.2%) |
| No | 4 (10.3%) |

Data are presented as n (%). ^a^Others include poorly, scirrhous, and adenosquamous; SSPPD, subtotal stomach-preserving pancreaticoduodenectomy; AJCC, American Joint Committee on Cancer; CA19-9, carbohydrate antigen 19-9.

**Supplementary Table S1b.** Characteristics of patients who did not undergo surgery

| Characteristics | Value |
| --- | --- |
|  | (N=39) |
| Sex |  |
| Male | 16 (41.0%) |
| Female | 23 (59.0%) |
| Age (median, 69.5 years) |  |
| >70 years | 21 (53.8%) |
| ≤70 years | 18 (46.2%) |
| AJCC stage |  |
| Stage III | 13 (33.3%) |
| Stage IV | 22 (56.4%) |
| Baseline CA19-9 level |  |
| ≥37 U/mL | 11 (28.2%) |
| <37 U/mL | 28 (71.8%) |
| Treatment |  |
| Chemotherapy-naïve status | 26 (66.7%) |
| Chemotherapy | 8 (20.5%) |
| Palliative treatment | 5 (12.8%) |
| First-line chemotherapy regimen |  |
| FOLFIRINOX | 8 (20.5%) |
| Gemcitabine+nab-paclitaxel | 19 (48.7%) |
| Gemcitabine | 4 (10.3%) |
| Gemcitabine+S1 | 2 (5.1%) |

Data are presented as n (%). AJCC, American Joint Committee on Cancer; CA19-9, carbohydrate antigen 19-9; FOLFIRINOX, folinic acid, fluorouracil, irinotecan, and oxaliplatin.
