# Supplementary material for: Longitudinal monitoring of KRAS-mutated circulating tumor DNA enables the prediction of prognosis and therapeutic responses in patients with pancreatic cancer
Source: PLoS One. 2019 Dec 31;14(12):e0227366. doi: 10.1371/journal.pone.0227366 (PMC6938323; doi:10.1371/journal.pone.0227366)
Supplement: S2 Table — (DOC) [file pone.0227366.s007.doc]

**Supplementary Table S2.** Clinical information of patients who underwent surgery

| Patients | Sex | Age | Tumor location | Operation methods | AJCC stage | Pathological differentiation | *KRAS* mutation | | Preoperative CA19-9 level | Emergence of *KRAS*-mutated ctDNA | | NAC | Adjuvant chemotherapy | Recurrence |
| --- | --- | --- | --- | --- | --- | --- | --- | --- | --- | --- | --- | --- | --- | --- |
|  |  | (years) |  |  |  |  | Primary tissue by RASKET | Primary tissue by ddPCR |  | Before surgery | In monitoring |  |  |  |
| 1 | m | 49 | Tail | DP | IIA | Well | 12V | 12V, 12D | 117.3 |  |  | No | S1 | No |
| 2 | m | 78 | Body | DP | IIB | Well | 12R | 12R | 178.7 |  |  | No | S1 | No |
| 3 | f | 70 | Body | DP | IIB | Moderately | 12D | 12D, 12V | 54.9 | 12D |  | No | S1 | No |
| 4 | f | 58 | Head, body | TP | IIB | Well | 12V | 12V, 12D | 162.3 |  |  | No | S1 | No |
| 5 | f | 81 | Head | SSPPD | IIB | Well | 12D | 12D, 12V, 12R | 0.7 |  |  | No | S1 | No |
| 6 | m | 67 | Head | SSPPD | IIB | Well | Wild | 12D | 511.8 |  |  | No | S1 | No |
| 7 | m | 69 | Head | SSPPD | IIB | Well | Wild | 12D, 12V | 94 | ND |  | No | S1 | Local |
| 8 | f | 67 | Body | DP | IIB | Well | 12D | 12D | 91.7 | 12D |  | No | S1 | No |
| 9 | f | 69 | Head | TP | IA | Well | Q61H | Q61H, 12D, 12V | 36.4 |  |  | No | S1 | No |
| 10 | m | 75 | Head | SSPPD | IIB | Well | 12V | 12V, 12D | 256.6 |  |  | No | No | No |
| 11 | m | 66 | Tail | DP | IIA | Adenosquamous | 12D | 12D | 11.5 | 12D |  | No | S1 | No |
| 12 | m | 69 | Head | SSPPD | IIB | Well | 12V | 12V | 27.5 | ND |  | No | S1 | No |
| 13 | m | 79 | Tail | Lap-DP | IIB | Well | 12D | 12D | 76.1 |  |  | No | No | No |
| 14 | m | 72 | Head | SSPPD | IB | Moderately | 12D | 12D | 717.4 | 12D |  | No | S1 | No |
| 15 | f | 64 | Head | TP | IIB | Well | Wild | 12V | 43.5 |  |  | GnP | S1 | No |
| 16 | f | 63 | Body | DP | IIB | Well | 12D | 12D, 12V | 27.1 | ND |  | No | S1 | Lung |
| 17 | f | 67 | Head | SSPPD | IIB | Well | 12V | 12V | 16160 |  |  | No | Gemcitabine | Local |
| 18 | m | 67 | Head | SSPPD | IIA | Well | 12V | 12V, 12D | 10.7 | ND | 12V | FOLFIRINOX | No | Local |
| 19 | f | 73 | Body | DP | IIB | Well | Wild | 12D, 12V | 19.4 | ND |  | GS | S1 | Lymph node |
| 20 | f | 75 | Head | SSPPD | IIB | Well | 12D | 12D, 12R | 2841.3 |  |  | No | S1 | Lymph node |
| 21 | m | 77 | Body, tail | DP | IIA | Well | 12V | 12V, 12D | 816.1 | ND |  | No | No | Liver |
| 22 | m | 73 | Tail | DP | IIA | Well | 12V | 12V, 12D | 893.7 | 12V | 12V | No | S1 | Lung |
| 23 | f | 72 | Head, body | TP | IIB | Well | 12V | 12V, 12R | 107.5 | ND |  | No | Gemcitabine | Peritoneum |
| 24 | f | 69 | Head | SSPPD | IV | Moderately | 12D | 12D, 12V | 2853.4 |  |  | No | S1 | Peritoneum |
| 25 | m | 73 | Head | SSPPD | IIA | Well | ND | 12R, 12D | 100.2 |  |  | GnP | S1 | Lymph node, peritoneum |
| 26 | m | 72 | Head | SSPPD | IIA | Moderately | 12V | 12V, 12D | 2986 | 12V |  | No | S1 | No |
| 27 | f | 76 | Body, tail | Lap-DP | IIB | Moderately | 12D | 12D, 12V | 4.9 |  |  | No | S1 | No |
| 28 | m | 76 | Head | SSPPD | IIB | Well | 12D | 12D | 128.8 | ND | 12D | No | No | No |
| 29 | f | 66 | Head | SSPPD | IIA | Well | 12V | 12V, 12D | 400.7 |  | 12V, 12R | No | S1 | Liver, local |
| 30 | m | 43 | Body | DP | IA | Poorly | 12D | 12D | 13.7 |  | 12D | No | S1 | Liver, local |
| 31 | f | 74 | Head | SSPPD | IIB | Moderately | 12D | 12D, 12V | 103 |  | 12D | GS | S1 | Liver |
| 32 | f | 83 | Head | SSPPD | IIB | Well | 12V | 12V, 12D | 1875 | ND | 12V | No | S1 | Bone |
| 33 | m | 70 | Body | DP | IIA | Well | 12D | 12D | 296.8 |  | 12D | No | S1 | Liver |
| 34 | f | 66 | Body | DP | IIB | Moderately | 12V | 12V, 12D | 222.3 |  | 12V | No | S1 | Liver |
| 35 | f | 79 | Head, body | TP | IIB | Moderately | 12V | 12V, 12D | 346.8 |  | 12V | No | S1 | Peritoneum |
| 36 | m | 70 | Head, uncus | SSPPD | IIA | Moderately | 12D | 12D | 943.7 |  | 12D | No | S1 | Lymph node |
| 37 | m | 56 | Head | SSPPD | IIB | Scirrhous | 12V | 12V, 12D | 11.1 |  | 12V | No | S1 | Liver |
| 38 | m | 66 | Body | DP-CAR | III | Moderately | 12D | 12D | 570.4 | 12D | 12D | GnP | No | Lung, liver |
| 39 | m | 70 | Head | SSPPD | IIA | Well | Wild | 12D | 19.1 |  | 12D | GnP | S1 | Local, lung, lymph node |

ddPCR, droplet digital polymerase chain reaction; CA19-9, carbohydrate antigen 19-9; ctDNA, circulating tumor DNA; NAC, neoadjuvant chemotherapy; DP, distal pancreatectomy; TP, total pancreatectomy; SSPPD, subtotal stomach-preserving pancreaticoduodenectomy; Lap-DP, laparoscopic distal pancreatectomy; DP-CAR, distal pancreatectomy with celiac axis resection; AJCC, American Joint Committee on Cancer; ND, not determined; Local, recurrence of residual pancreas; FOLFIRINOX, folinic acid+fluorouracil+irinotecan+oxaliplatin; GnP, gemcitabine+nab-paclitaxel; GS, gemcitabine+S1; blank, no detection of *KRAS*-mutated ctDNA.
