# Supplementary material for: Longitudinal monitoring of KRAS-mutated circulating tumor DNA enables the prediction of prognosis and therapeutic responses in patients with pancreatic cancer
Source: PLoS One. 2019 Dec 31;14(12):e0227366. doi: 10.1371/journal.pone.0227366 (PMC6938323; doi:10.1371/journal.pone.0227366)
Supplement: S3 Table — (DOC) [file pone.0227366.s008.doc]

**Supplementary Table S3.** Clinical information of patients who did not undergo surgery

| Patients | Sex | Age | Unresectable factor | *KRAS* mutation | | CA19-9 level before first-line chemotherapy | Emergence of *KRAS*-mutated ctDNA before first-line chemotherapy | Emergence of *KRAS-* mutated ctDNA | Treatments | Chemotherapy |
| --- | --- | --- | --- | --- | --- | --- | --- | --- | --- | --- |
|  |  | (years) |  | In tissue by RASKET | In tissue by ddPCR |  |  | In monitoring |  | First-line |
| 40 | f | 46 | Recurrence (liver) | Wild | 12D, 12R | NA | NA | 12D | Treated | NA |
| 41 | m | 56 | Local advanced | 12V | 12V | 456.8 |  |  | Treatment-naïve | FOLFIRINOX |
| 42 | f | 65 | Local advanced | ND | 12V, 12D | 132 | 12V | 12V | Treatment-naïve | GnP |
| 43 | f | 65 | LN metastasis | 12V | 12V, 12D | 11.1 | 12V | 12V | Treatment-naïve | FOLFIRINOX |
| 44 | m | 76 | Local advanced, lung metastasis | 12V | 12V | 233.5 |  | 12V | Treatment-naïve | GnP |
| 45 | f | 69 | Local advanced | 12R | 12R, 12D | 114.5 |  |  | Treatment-naïve | GnP |
| 46 | m | 67 | Local advanced | ND | 12D | 1205.2 |  |  | Treatment-naïve | Gemcitabine |
| 47 | m | 46 | Recurrence (liver, residual pancreas) | 12D | 12D | 218.3 |  |  | Treatment-naïve | FOLFIRINOX |
| 48 | f | 76 | Para-aortic LN metastasis | Wild | 12D | 6.8 | 12D | 12D | Treatment-naïve | GnP |
| 49 | f | 44 | Local advanced | 12D | 12D | 174.9 |  |  | Treatment-naïve | FOLFIRINOX |
| 50 | f | 74 | Liver, lung metastasis | 12R | 12R, 12D | 47.2 |  |  | Treatment-naïve | GnP |
| 51 | f | 81 | LN metastasis | 12V | 12V, 12D | 423.3 |  |  | Treatment-naïve | GnP |
| 52 | m | 62 | Local advanced, LN metastasis | 12V | 12V, 12D | 700 |  | 12V | Treatment-naïve | FOLFIRINOX |
| 53 | f | 75 | Recurrence (lung, residual pancreas) | Wild | 12D | NA | NA |  | Treated | NA |
| 54 | m | 65 | Liver metastasis | ND | ND | 62 | 13D | 13D | Treatment-naïve | GnP |
| 55 | m | 63 | Peritoneal dissemination | 12R | 12R, 12D | 14840 | 12R | 12R | Treatment-naïve | FOLFIRINOX |
| 56 | f | 75 | Liver metastasis | Q61H | Q61H | NA | NA | Q61H | Treated | NA |
| 57 | f | 74 | Local advanced, LN metastasis, peritoneal dissemination | 12R | 12R, 12D | 112.2 |  | 12D | Treatment-naïve | GnP |
| 58 | f | 65 | Local advanced | 12V | 12V | 4.9 | 12V | 12V | Treatment-naïve | GnP |
| 59 | f | 84 | Local advanced | ND | 12V, 12D | 4223.4 | 12V | 12D, 12V | Treatment-naïve | Gemcitabine |
| 60 | f | 40 | Liver, lung metastasis | ND | 12D | 15276.9 |  | 12D | Treatment-naïve | FOLFIRINOX |
| 61 | m | 72 | Local advanced | ND | ND | 576 |  | 12D | Treatment-naïve | GnP |
| 62 | m | 68 | Local advanced | 12D | 12D, 12R | NA | NA | 12D | Treated | NA |
| 63 | f | 74 | Local advanced | Q61H | Q61H, 12D | 23.1 |  |  | Treatment-naïve | GnP |
| 64 | f | 84 | Local advanced | ND | ND | NA | NA |  | Treated | NA |
| 65 | f | 62 | LN metastasis | 12D | 12D | NA | NA | 12D | Treated | NA |
| 66 | m | 65 | Liver metastasis | 12V | 12V | 1518 |  | 12V | Treatment-naïve | GnP |
| 67 | f | 72 | Liver metastasis | 12D | 12D | 2024 | 12D | 12D | Treatment-naïve | GnP |
| 68 | m | 70 | Liver metastasis, peritoneal dissemination | 12D | 12D | 1795.4 | 12D | 12D | Treatment-naïve | GnP |
| 69 | m | 68 | Liver, lung metastasis, peritoneal dissemination | 12V | 12V | 32664.5 | 12V | 12V | Treatment-naïve | GnP |
| 70 | f | 66 | Peritoneal dissemination | 12V | 12V, 12D | NA | NA | 12V | Palliative treatment | NA |
| 71 | f | 71 | Recurrence of residual pancreas | 12R | 12R, 12D, | NA | NA |  | Treated | NA |
| 72 | m | 69 | Liver metastasis | Q61H | Q61H | 1213.7 | Q61H | Q61H | Treatment-naïve | GnP |
| 73 | m | 72 | Liver, lung metastasis | ND | 12D | 7700 | 12D | 12D | Treatment-naïve | GnP |
| 74 | f | 78 | Local advanced | 12V | 12V | NA | NA | 12V | Treated | NA |
| 75 | m | 55 | Local advanced | ND | ND | NA | NA | 12D | Palliative treatment | NA |
| 76 | f | 62 | Liver metastasis | 12V | 12V, 12D | NA | NA | 12V | Palliative treatment | NA |
| 77 | f | 60 | Liver, lung metastasis, local advanced | 12D | 12D, | NA | NA | 12D | Palliative treatment | NA |
| 78 | m | 74 | Liver, lung metastasis | ND | ND | NA | NA | 12D | Palliative treatment | NA |

ddPCR, droplet digital polymerase chain reaction; CA19-9, carbohydrate antigen 19-9; ctDNA, circulating tumor DNA; LN, lymph node; ND, not determined; NA, not applicable; FOLFIRINOX, folinic acid+fluorouracil+irinotecan+oxaliplatin; GnP, gemcitabine+nab-paclitaxel; blank, no detection of *KRAS*-mutated ctDNA.
