# Supplementary material for: Longitudinal monitoring of KRAS-mutated circulating tumor DNA enables the prediction of prognosis and therapeutic responses in patients with pancreatic cancer
Source: PLoS One. 2019 Dec 31;14(12):e0227366. doi: 10.1371/journal.pone.0227366 (PMC6938323; doi:10.1371/journal.pone.0227366)
Supplement: Attachment — Submitted filename: CTDNAPDACoutcomes.pdf [file pone.0227366.s010.pdf]

Author Manuscript

Author Manuscript

Author Manuscript

Author Manuscript

HHS Public Access

Author manuscript

Gastroenterology. Author manuscript; available in PMC 2019 March 26.

Published in final edited form as:

Gastroenterology. 2019 January ; 156(1): 108–118.e4. doi:10.1053/j.gastro.2018.09.022.

Circulating Nucleic Acids Are Associated With Outcomes of Patients With Pancreatic Cancer

Vincent Bernard[1,2,*], **Dong U. Kim**[1,3,*], **F. Anthony San Lucas**[4], **Jonathan Castillo**[1], **Kelvin Allenson**[1,5], **Feven C. Mulu**[1], **Bret M. Stephens**[1], **Jonathan Huang**[1], **Alexander Semaan**[1], **Paola A. Guerrero**[1], **Nabiollah Kamyabi**[1], **Jun Zhao**[1], **Mark W. Hurd**[6], **Eugene J. Koay**[7], **Cullen M. Taniguchi**[7], **Joseph M. Herman**[7], **Milind Javle**[8], **Robert Wolff**[8], **Matthew Katz**[5], **Gauri Varadhachary**[8], **Anirban Maitra**[1,6], and **Hector A. Alvarez**[9]

[1]Department of Pathology, The University of Texas MD Anderson Cancer Center, Houston, Texas

[2]The University of Texas MD Anderson Cancer Center UTHealth Graduate School of Biomedical Sciences, Houston, Texas

[3]Department of Internal Medicine, Biomedical Research Institute, Pusan National University Hospital, Pusan National University School of Medicine, Busan, Korea

[4]Department of Epidemiology, The University of Texas MD Anderson Cancer Center, Houston, Texas

[5]Department of Surgical Oncology, The University of Texas MD Anderson Cancer Center, Houston, Texas

[6]Sheikh Ahmed Pancreatic Cancer Research Center, The University of Texas MD Anderson Cancer Center, Houston, Texas

[7]Department of Radiation Oncology, The University of Texas MD Anderson Cancer Center, Houston, Texas

[8]Department of Gastrointestinal Medical Oncology, The University of Texas MD Anderson Cancer Center, Houston, Texas

[9]Department of Hematopathology, The University of Texas MD Anderson Cancer Center, Houston, Texas

Abstract

BACKGROUND & AIMS: We aimed to investigate the clinical utility of circulating tumor cell DNA (ctDNA) and exosome DNA (exoDNA) in pancreatic cancer.

Reprint requests Address requests for reprints to: Hector A. Alvarez, MD, 6565 MD Anderson Boulevard Z3.3012, Houston, Texas 77030. Haalvarez@mdanderson.org; fax: (713) 745-0838.
[*]Authors share co-first authorship.

Conflicts of interest
The authors disclose no conflicts.

Supplementary Material
Note: To access the supplementary material accompanying this article, visit the online version of *Gastroenterology* at www.gastrojournal.org, and at https://doi.org/10.1053/j.gastro.2018.09.022.

Author Manuscript
Author Manuscript
Author Manuscript
Author Manuscript

METHODS: We collected liquid biopsy samples from 194 patients undergoing treatment for localized or metastatic pancreatic adenocarcinoma from April 7, 2015, through October 13, 2017 (425 blood samples collected before [baseline] and during therapy). Additional liquid biopsy samples were collected from 37 disease control individuals. Droplet digital polymerase chain reaction was used to determine *KRAS* mutant allele fraction (MAF) from ctDNA and exoDNA purified from plasma. For the longitudinal analysis, we analyzed exoDNA and ctDNA in 123 serial blood samples from 34 patients. We performed analysis including Cox regression, Fisher exact test, and Bayesian inference to associate *KRAS* MAFs in exoDNA and ctDNA with prognostic and predictive outcomes.

RESULTS: In the 34 patients with potentially resectable tumors, an increase in exoDNA level after neoadjuvant therapy was significantly associated with disease progression (P = .003), whereas ctDNA did not show correlations with outcomes. Concordance rates of *KRAS* mutations present in surgically resected tissue and detected in liquid biopsy samples were greater than 95%. On univariate analysis, patients with metastases and detectable ctDNA at baseline status had significantly shorter times of progression-free survival (PFS) (hazard ratio [HR] for death, 1.8; 95% CI, 1.1–3.0; P = .019), and overall survival (OS) (HR, 2.8; 95% CI, 1.4–5.7; P = .0045) compared with patients without detectable ctDNA. On multivariate analysis, MAFs ≥5% in exoDNA were a significant predictor of PFS (HR, 2.28; 95% CI, 1.18–4.40; P = .014) and OS (HR, 3.46; 95% CI, 1.408.50; P = .007). A multianalyte approach showed detection of both ctDNA and exoDNA MAFs ≥5% at baseline status to be a significant predictor of OS (HR, 7.73, 95% CI, 2.61–22.91, P = .00002) on multivariate analysis. In the longitudinal analysis, an MAF peak above 1% in exoDNA was significantly associated with radiologic progression (P = .0003).

CONCLUSIONS: In a prospective cohort of pancreatic cancer patients, we show how longitudinal monitoring using liquid biopsy samples through exoDNA and ctDNA provides both predictive and prognostic information relevant to therapeutic stratification.

Graphical Abstract

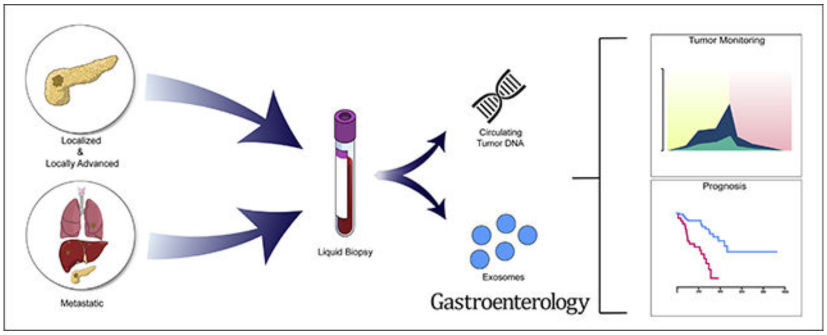

Keywords

PDAC; Extracellular Vesicles; Biomarkers; Tumor Monitoring

Although rare, pancreatic ductal adenocarcinoma (PDAC) has recently become the third-leading cause of cancer-related deaths, with projections of it rising to the second-leading cause of cancer deaths within the next decade.[1] Although surgical resection provides a potential curative option in PDAC, only a minority of patients (<15%) will be diagnosed

Author Manuscript Author Manuscript Author Manuscript Author Manuscript

with disease that is amenable to surgery, and even in this subset of patients, 5-year overall survival (OS) rates remain below 30%. Neoadjuvant therapies are increasingly being adopted to enhance local disease control in patients with resectable tumors. Because most PDAC patients present with surgically unresectable tumors, current therapeutic options in this patient population has resulted in modest benefits in OS with no current means to personalize therapy. Among patients with both localized and metastatic disease, there still remains a significant unmet need in developing more effective strategies for therapeutic stratification and management.

The use of blood-based biomarkers for cancer diagnosis and therapeutic stratification has gained significant traction in cancer in the form of circulating proteins, RNA, and DNA. Specifically, circulating tumor DNA (ctDNA) detection in the blood of breast, colorectal, and lung cancer patients, among others, has shown clinical relevance in identifying patient relapses.[2–6] In the context of PDAC, the use of ctDNA as a clinically significant biomarker has been inconsistent with regard to its prognostic and predictive potential.[7–11] Additional sources of DNA and RNA in circulation have been recently identified in the form of microvesicles known as *exosomes*.[12] Previous studies have shown the utility of profiling the genomic content of exosomes (exoDNA) as a surrogate for the mutational landscapes of established cancers and for early detection. [11,13,14] These 40–150-nm lipid bilayer membrane-bound vesicles are believed to form protective barriers of nucleic acid material from nuclease-induced degradation in the plasma, thus allowing for the native material to exist in a high-molecular-weight format compared with ctDNA, which is mostly found at 170 base pairs. This could allow for greater resolution and sensitivity of molecular profiling of high-quality DNA material.

In this study, we aimed to compare the utility of tumor monitoring in patients with localized and metastatic PDAC using paired exoDNA and ctDNA to determine how they may be used in a complementary manner for prognostication and therapeutic stratification. We performed longitudinal collection in a large prospective cohort of PDAC patients with localized and metastatic cancer, such that the dynamics of *KRAS* mutation detection in circulation could be correlated with disease progression and compared with standard readouts, such as imaging and carbohydrate antigen (CA) 19–9. To our knowledge, this study represents the first comprehensive comparison of these liquid biopsy compartments in the context of clinical utility. Additionally, we believe that the longitudinal aspects of this study have implications for potential real-time therapeutic stratification of PDAC patients.

Materials and Methods

Study Design

Patients with clinically and histologically confirmed localized or metastatic pancreatic adenocarcinoma, defined by American Joint Committee on Cancer guidelines,[15] were enrolled in this longitudinal cohort study. Metastatic disease was based on surgical or radiologic confirmation. A total of 194 patients were recruited at MD Anderson Cancer Center and gave informed consent after institutional review board approval (PA14–0552 and PA11–0670); they were treated between April 7, 2015, and October 13, 2017 (Figure 1, Supplementary Figure 1, and Supplementary Table 1). Of these, 104 patients presented at

Author Manuscript Author Manuscript Author Manuscript Author Manuscript

baseline with metastatic PDAC and treatment-naïve status. An additional 25 patients with pancreatic cysts were recruited and classified based on computed tomographic (CT) imaging. If receiving first-line therapy, treatment-naïve patients underwent pretreatment CT imaging and were followed up every 2–3 months with restaging imaging after initiation of chemotherapy. Progression in all patients was determined based on routine clinical evaluation by a blinded, board-certified radiologist based on RECIST 1.1 criteria of CT imaging.[16] PFS was defined by the time from start of first-line therapy to progression based on CT restaging imaging.

Blood Sample Collection and Processing

A total of 318 blood samples were prospectively acquired from patients with metastatic disease at MD Anderson Cancer Center. Approximately 25 mL of whole peripheral blood was collected in acid citrate dextrose tubes (BD Biosciences) and processed within 2 hours of phlebotomy. Blood samples were centrifuged at 2500*g* for 10 minutes for plasma isolation and were aliquoted accordingly for downstream processing, where 1 mL was used for ctDNA isolation and approximately 12 mL was used for exosome isolation. Exosome and ctDNA isolations were performed fresh in all samples.

Exosome Isolation

Exosomes were isolated from plasma as previously described.[12] Briefly, plasma aliquots were diluted in approximately 35 mL of phosphate-buffered saline (PBS) at 4°C. Samples were centrifuged at 1000 revolutions/minute for 5 minutes at 4°C to remove cells, and then supernatant was recovered and centrifuged at 5000 revolutions/minute for 10 minutes at 4°C to remove cell debris and platelets. Supernatant was further diluted in PBS and ultracentrifuged at 154,000*g* overnight, with a second ultracentrifugation wash step of 2 hours, both at 4°C. The resulting exosome pellet was resus-pended in PBS and harvested for downstream analyses.

ExoDNA and ctDNA Extraction

ctDNA and exoDNA were isolated using the QIAamp Circulating Nucleic Acid Kit (Qiagen) according to the manufacturer's protocol, as previously described.[14]

Digital Droplet PCR Analysis

Droplet digital polymerase chain reaction (ddPCR) (QX200; BioRad) was used for highly sensitive detection of genetic mutations with a multiplex *KRAS* assay containing G12V, G12D, G12R, G12C, G12S, G12A, and G13D mutant codons, as previously described.[11,14]

Results

Characteristics of Patients Undergoing Liquid Biopsies

Study overview and patient stratification are presented in Supplementary Figure 1 and Supplementary Tables 1 and 2. A total of 318 blood samples from 123 patients with metastatic disease and 107 blood samples from 71 patients with localized resectable tumors were profiled with ddPCR. Median follow-up time for all patients was 187 days. ExoDNA

Author Manuscript Author Manuscript Author Manuscript Author Manuscript

and ctDNA profiled at baseline treatment-naïve status showed *KRAS* mutation detection rates of 61% and 53%, respectively, in patients with metastatic disease and 38% and 34%, respectively, in patients with localized disease (Figure 1A and B). To determine the prevalence of circulating mutational events in other pancreatic diseases, an additional 37 patients with pancreatic lesions were evaluated for *KRAS* mutations in exoDNA and ctDNA (Supplementary Table 3). Mutation detection rates were 12% (3/25) and 16% (4/25) for pancreatic cysts and 25% (3/12) and 17% (2/12) for nonneoplastic pancreatic disease for exoDNA and ctDNA, respectively. When all patient populations were compared, those with metastatic disease had significantly greater circulating mutant allelic fractions (MAFs) of *KRAS* compared with patients with localized disease and pancreatic cysts (Figure 1C and Supplementary Table 2). Patients with localized disease had significantly greater MAF than patients with pancreatic cysts. We also determined criterion standard validation of concordance rates between exoDNA and ctDNA with tumor tissue for *KRAS* mutation detection using ddPCR (Supplementary Table 4). Concordance among 22 surgically resected primary pancreatic tumors was 95.5% and 68.2% for exoDNA and ctDNA, respectively, whereas concordance from 12 samples derived by fine needle aspirates was 83.3% and 66.8% for exoDNA and ctDNA, respectively.

Serial Liquid Biopsy Results in Patients With Localized PDAC Receiving Neoadjuvant Therapy Are Predictive of Eventual Surgical Resection

A total of 34 PDAC patients with localized disease were serially monitored during neoadjuvant therapy, comprising 68 cumulative blood draws taken at baseline and after the completion of neoadjuvant therapy (Supplementary Table 5). The kinetics of circulating *KRAS* mutational burden were then measured in exoDNA and ctDNA with ddPCR. Mutant *KRAS* was detected at baseline in 41% (14/34) and 32% (11/34) of patients in exoDNA and ctDNA, respectively. Among the patients monitored, 50% (17/34) had subsequent surgical resection given an absence of disease progression, compared with 50% who experienced disease progression, primarily manifesting as the emergence of new metastatic lesions. In this cohort, reduction in exo*KRAS* MAF from baseline at the completion of neoadjuvant therapy was significantly correlated with surgical resection, and the reverse was true for patients who did not emerge as surgical candidates (odds ratio [OR], 38.4; 95% confidence interval [CI], 3.95–373.3; $P = .0002$) (Figure 2A and B). Specifically, among patients who had resection, 71% (12/17) experienced a decrease in exo*KRAS* MAF from baseline treatment-naïve values, and in those patients who did not, 16 of 17 (94%) saw an increase or no change in *KRAS* MAF in exoDNA from baseline status. As an example, in an index case, a rise in *KRAS* MAF in exoDNA suggested progressive disease, although that was initially not detectable by CA19–9 or CT imaging. On laparotomy, CT-occult omental metastasis was found, resulting in an aborted resection. This correlation between changes in *KRAS* MAF and resectability, however, was not seen with ctDNA. After eliminating those patients who were considered as nonexpressors of CA19–9 (values <37 U/mL), changes in CA19–9 were also significantly correlated with those patients likely to have surgery (OR, 28.0; CI, 2.65–295.9; $P = .003$). Among 3 patients for whom no detectable *exoKRAS* mutant was found, CA19–9 was able to predict progressive disease, underlining the complementary nature of how these biomarkers can be used.

Author Manuscript Author Manuscript Author Manuscript Author Manuscript

Clinical Correlates of Liquid Biopsy Samples at Presentation in Patients With Metastatic PDAC

Among patients with metastatic PDAC, clinical characteristics at the time of presentation were grouped according to exoDNA and ctDNA status (Supplementary Table 2). There was no significant association between *KRAS* MAF in exoDNA and ctDNA and presenting characteristics. Overall, 66 (63%) had experienced radiologic progression, and 69 (67%) were still alive at last follow-up date. Patients who experienced progression during serial monitoring or succumbed to disease had higher *KRAS* MAF in exoDNA at presentation (Wil- coxon signed-rank tests, $P = .03$ and $P = .01$, respectively) compared with those whose disease had not progressed (Supplementary Figure 2A). ctDNA tumor burden, as measured by *KRAS* MAF at presentation, was also significantly associated with survival ($P = .03$) (Supplementary Figure 2B). Patients with liver metastatic lesions had a significantly greater *KRAS* MAF in exoDNA and ctDNA than patients with isolated lung and peritoneal lesions ($P = .04$) (Supplementary Figure 3A and B). This correlation was likely affected by the fact that patients with metastasis to the liver have larger volume of lesions than those with isolated lung and peritoneal metastases (Supplementary Figure 3C). In fact, on linear regression analysis, exoDNA and ctDNA *KRAS* MAFs at presentation were significantly correlated with tumor size as measured by total sum of lesion diameters ($P = .035$ and $P = .0008$, respectively) (Supplementary Figure 4). Additionally, patients with progressively worse ECOG performance status harbored significantly greater *KRAS* MAF (Supplementary Figure 5).

Prognostic Impact of Liquid Biopsy Parameters at Presentation in Patients With Metastatic PDAC

To avoid confounding effects of chemotherapy on exoDNA and ctDNA kinetics, we performed subset analysis on 104 metastatic patients who were treatment naïve at the time of presentation. An optimal threshold for ctDNA was assessed by first performing receiver operating curve analysis to estimate ideal cutoffs. As previously described in other tumor types, the presence and absence of detectable ctDNA (ie, any mutant *KRAS* on ddPCR) was significantly associated with patient outcomes.[2,5] For example, any detectable ctDNA was associated with significantly shorter PFS (log-rank test: HR, 1.93; 95% CI, 1.15–3.22; $P = .012$) with a median PFS of 118 vs 321 days (for detection vs no detection, respectively) (Figure 3B). Detectable ctDNA also showed shorter OS (HR, 2.36; 95% CI, 1.16–4.79; $P = .018$) with a median OS of 258 vs 440 days (detection vs no detection, respectively) (Figure 3B). In the context of exoDNA, an optimal *exoKRAS* MAF was determined to be 5%. Using this threshold of 5% *KRAS* MAF, 5% *KRAS* MAF >5% was significantly associated with reduced PFS (HR, 4.78; 95% CI, 2.47–9.26; $P < .0001$) and OS (HR, 7.31; 95% CI, 3.15–17.00; $P < .0001$) on Kaplan-Meier analysis (Figure 3A). Similarly, survival analyses of the standard clinical biomarker CA19–9 (Supplementary Figure 6) showed that patients with a CA19–9 ≥300 U/mL at treatment-naïve presentation had worse OS ($P = .023$), with PFS trending toward significance ($P = .06$).

With a Cox regression model (Supplementary Table 6), univariate analysis showed that *KRAS* MAF ≥5% in exoDNA (HR, 3.5; 95% CI, 2.1–5.9; $P < .0001$) and any ctDNA detection (HR, 1.8; 95% CI, 1.1–3.0; $P = .019$) were significantly associated with shorter

Author Manuscript

PFS. On multivariate analysis, exoKRAS ≥5% remained the only significant predictor of PFS (HR, 2.28; 95% CI, 1.18–4.40; *P* = .014). Combining *KRAS* MAF ≥5% in exoDNA or ctDNA detection with a CA19–9 ≥300 did not show an increase in predictive significance of these biomarkers for poorer PFS (Supplementary Table 7).

For OS, *KRAS* MAF ≥5% in exoDNA (HR, 4.6; 95% CI, 2.2– 9.7; *P* < .0001), any ctDNA detection (HR, 2.8; 95% CI, 1.4–5.7; *P* = .0045), CA19–9 ≥300 U/mL (HR, 3.2; 95% CI, 1.3–7.7; *P* = .011), and an ECOG performance status score of 2 (HR, 3.56; 95% CI, 1.04–12.3; *P* = .044) were significant predictors of poorer outcomes on univariate analysis. On multivariate analysis, exoKRAS ≥5% (HR, 3.46; 95% CI, 1.40–8.50; *P* = .007) remained as a significant predictor of poorer OS. An *exoKRAS* MAF ≥5% together with a CA19–9 ≥300 U/mL (HR, 6.41; 95% CI, 2.31–17.80; *P* = .0004) at baseline treatment-naïve status was a significant predictor of poorer OS. Although on its own ctDNA did not emerge as a significant predictor on multivariate analysis, detection of ctDNA emerged as a significant predictor of poorer OS when it occurred with a CA19–9 ≥300 U/mL at baseline treatment-naïve status (HR, 6.37; 95% CI, 2.36–17.24; *P* = .0003). Additionally, exoKRAS MAF ≥5% and ctDNA detection were correlated to poorer OS (HR, 7.73; 95% CI, 2.6122.91; *P* = .00002) on multivariate analysis when both occurring at baseline treatment-naïve status in the same patient, underlining the potential complementary nature of these biomarkers.

Longitudinal Monitoring of Metastatic PDAC With Serial Liquid Biopsy Samples Anticipates On-Treatment Progression

To fully evaluate the utility of liquid biopsies for monitoring the natural history of metastatic PDAC, we profiled exoDNA and ctDNA through 123 serial blood draws from 34 patients with a median follow-up time of 202 days (Supplementary Figure 1A). Specifically, we selected patients who had at least 2 blood draws taken during a concurrent therapeutic regimen, with 2 or more restaging imagings taken at standard 2–3-month intervals. Among the monitored patients, disease progressed in 20 of 34 (59%) while receiving therapy, with a median time to progression of 176 days. Patients whose disease did not progress had a median follow-up time of 300 days. Analysis of plasma samples showed that a *KRAS* MAF peak of ≥1% in any on- treatment serial exoDNA sample was significantly associated with eventual disease progression, as determined by RECIST 1.1 *(P* < .0001) (Figure 4A and B). The optimal MAF of ctDNA *KRAS* and exoDNA *KRAS* in predicting progression was assessed by receiver operating curve analysis, with only exo*KRAS* achieving predictive significance, with sensitivity and specificity of 79% and 100%, respectively. Among the 20 patients whose disease progressed, 16 (80%) saw an exoDNA *KRAS* MAF peak of ≥1% compared with none of those patients without progression 14/14 (100%). In contrast, serial ctDNA MAF did not correlate significantly with presence or absence of progression. With a threshold of 20% or greater increase of CA19–9 during therapy, the sensitivity and specificity of CA19–9 in predicting progression was 70% and 89%, respectively. When we assessed for the length of time when *KRAS* MAF in serial exoDNA exceeded ≥1% and the subsequent onset of radiologic progression, we found that exo*KRAS* had a significantly longer lead time (median of 50 days, *P* = .03) compared with lead times obtained by using 20% or greater increase in serial CA19–9 (which essentially coincided with the onset of radiologic progression) (Figure 4C). Additional application of Bayesian inference provided

Author Manuscript Author Manuscript Author Manuscript Author Manuscript

us with posterior probabilities of a 100% chance of progression given an *exoKRAS* peak ≥1% (P(Progression | *exoKRAS* ≥1%)) and 90% chance of prolonged response to therapy (no progression recorded before censoring) given that exo*KRAS* remains <1% (P(No Progression | exo*KRAS* <1%)).

Discussion

Nucleic acids derived from exosomes have been reported as a novel compartment of high-quality DNA material that is protected from degradation in circulation.[13,12,17] As opposed to ctDNA, which exists in the 150–170-base pair range, protected exoDNA is found in a high-molecular- weight format that readily lends itself to next-generation sequencing for molecular profiling. In our study, we profile-matched exoDNA and ctDNA for mutant *KRAS* alleles by quantitative ddPCR in a large series of prospectively collected plasma samples from PDAC patients (N = 194), and we identified baseline detection rates of 61% and 53% in metastatic disease and 38% and 34% in localized disease for exoDNA and ctDNA, respectively. A substantial minority of patients (12%−25%) with preneoplastic pancreatic cysts or nonneoplastic pancreatic diseases (such as chronic pancreatitis) harbored detectable circulating mutants. *KRAS* mutations are present in up to 80% of preneoplastic pancreatic cysts (including low-grade mucinous cysts),[18,19] and thus their detection on ddPCR in the circulation is not surprising. Mutations in *KRAS* are also detectable in the pancreas as a consequence of nonneoplastic inflammatory processes such as chronic pancreatitis, although tissue- based studies have confirmed a lower frequency of mutations than in either cancer or in preneoplastic cysts.[8,20,21] In line with these observations, and as an indirect derivation of tissue mutation load, quantitative ddPCR found average *KRAS* MAF to be highest in baseline metastatic samples, followed by localized disease, cystic lesions, and, finally, nonneoplastic pancreatic diseases, in that order.

Beyond detection of tumor-derived DNA per se in liquid biopsy samples as a biomarker of an underlying neoplasm, recent studies have also focused on the potential prognostic value imparted by ctDNA or exoDNA measurement in cancer patients at the time of presentation. For example, Mohrmann et al[22] reported that among 41 patients with advanced solid cancers, driver mutation detection by ddPCR in either exoDNA or ctDNA was associated with OS on Kaplan-Meier analysis, although only exoDNA at the time of presentation was an independent prognostic factor for OS on multivariate analysis (HR, 0.15; 95% CI, 0.03–0.80; $P = .026$). Along these lines, several studies have evaluated the prognostic potential of liquid biopsy samples in PDAC, most focusing on ctDNA measured with digital PCR. In a relatively small study, Earl et al[23] reported *KRAS* mutant ctDNA detection in 8 of 31 (26%) patients across various PDAC stages, with detection significantly correlated to OS (HR, 12.2; $P = .0002$). In a larger study of 105 patients, Hadano et al[24] reported a cumulative rate of 31% ctDNA detection across stages, with median survival of 13.6 months vs 27.6 months in those patients with detectable vs no detectable ctDNA, respectively, and a significant association with OS ($P < .0001$). In our own series, detection of ctDNA and exoDNA at presentation were both associated with significant deleterious impact on OS and PFS on univariate analysis, although only an exoDNA *KRAS* MAF ≥5% was an independent negative predictor of poor survival on multivariate analysis (HR, 2.28; 95% CI, 1.18–4.40; $P = .014$ for PFS and HR, 3.46; 95% CI, 1.40–8.50; $P = .007$ for OS, respectively) when used

Author Manuscript Author Manuscript Author Manuscript Author Manuscript

independently. Because previous work had shown the utility of using a combination of biomarkers for early detection, particularly ctDNA and CA19–9 in the context of surgically resectable pancreatic cancer, we aimed to determine the prognostic significance of combining ctDNA detection or exoDNA MAF with CA19–9.[25,26] Although ctDNA detection alone was not a significant predictor of outcomes, we did observe a combination of ctDNA detection and a CA19–9 level ≥300 U/mL at baseline treatment-naïve status to be a significant predictor of poorer OS (HR, 6.37; 95% CI, 2.36–17.24; $P = .0003$), showing the utility of a multianalyte approach. We also saw this same phenomenon when combining ctDNA detection and exo*KRAS* MAF ≥5% as a significant predictor of OS (HR, 7.73; 95% CI, 2.6122.91; $P = .00002$). Ultimately, these blood-based biomarkers show complementary utility in prognostic value, because the presence of these thresholds suggests that those patients may require more intense follow-up to capture earlier progression or more aggressive therapy than the standard of care to influence outcomes. This helps underline how each may represent distinct biologies, despite sharing the moniker of *liquid biopsy*, whereby ctDNA is released from apoptotic or necrotic cells, whereas exosomes may represent material released into circulation from rapidly dividing viable cells.

One major advantage of liquid biopsies is the ability to conduct longitudinal monitoring of on-treatment patients as a readout of therapeutic efficacy. Although this is typically conducted in PDAC with serial imaging scans or with CA19– 9, liquid biopsy samples might provide adjunctive, and potentially superior, predictive data on treatment response, with an opportunity for anticipating treatment intervention. In an earlier study, Tjensvoll et al[27] reported pilot data from a cohort of 14 PDAC patients, using peptide-nucleic acid- clamp PCR *KRAS* mutation detection. Monitoring ctDNA levels during chemotherapy showed a correlation with CA19–9 and radiologic progression in 3 patients. In a separate series, Sausen et al[28] used digital PCR for ctDNA detection after tumor resection in localized PDAC.[28] In 9 patients with detectable ctDNA and radiologic recurrence, the authors reported ctDNA detection an average of 3.1 months after resection compared with 9.6 months when it becomes clinically detectable on CT imaging. These data suggest a potential role for using liquid biopsies to facilitate earlier detection of progression than radiologic scans. In our cohort, we examined 34 metastatic patients who had sufficient longitudinal on-treatment follow-up and serial liquid biopsies to report tumor monitoring outcomes. Although we did not find significant association between progression outcomes with changes in ctDNA, we did find a significant correlation between exoDNA *KRAS* MAF and eventual radiologic progression. Specifically, those patients with an exoDNA *KRAS* MAF ≥1% on any on-treatment serial biopsy have a 100% probability of disease progression, with a median lead time to radiologic progression of 50 days from the first sample with exoDNA *KRAS* MAF ≥1%. In contrast, patients who maintained exoDNA *KRAS* MAF <1% on serial monitoring had a 90% probability of not progressing on therapy in the approximately 1-year median follow-up duration of our study. We believe that this mutant exoDNA spike to ≥1%, although transient, represents a growth spurt of the underlying cancer, likely coinciding with the incipient onset of resistance to ongoing therapy. The ability of serial liquid biopsies to predict which PDAC patients are most likely to fail first- or second-line chemotherapy is of clinical utility, because it provides an earlier opportunity than radiologic imaging for changing course. Continued exposure of patients to ineffective first- or second-line regimens

Author Manuscript Author Manuscript Author Manuscript Author Manuscript

may result in unnecessary toxicities and deterioration of performance status, which might make patients no longer candidates for subsequent therapies.

In addition to patients with metastatic disease, our prospective series also examined the utility of serial liquid biopsies in patients with localized PDAC. At MD Anderson, and increasingly at other centers in the United States, patients with localized disease receive preoperative (neoadjuvant) chemoradiation therapy. The main objective of neoadjuvant therapy is to prolong the survival of patients undergoing surgery and minimize the use of surgery for patients unlikely to benefit from it.[29] However, indicators of the effectiveness of neoadjuvant therapy and subsequent surgical resection remain a significant unmet need. We postulated that liquid biopsy kinetics between initiation and culmination of neoadjuvant treatment may predict response to neoadjuvant therapy and lack of progression, thus enabling surgery. In fact, a decrease in exoDNA *KRAS* MAF (but not ctDNA) between the beginning and the end of neoadjuvant therapy was significantly correlated with eventual surgical resection, when compared with those patients experiencing a rise in exoDNA *KRAS* MAF (OR, 38.4; 95% CI, 3.95–373.3; $P = .0002$). Although this same correlation held true for CA19–9 (OR, 28.0; 95% CI, 2.65–295.9; $P = .003$), which is not significantly different from exoDNA, it is worth noting how liquid biopsies may be used as complementary biomarkers in those patients who are deemed to be CA19–9 nonexpressors or those patients with obstructive jaundice, where CA19–9 shows no correlation to progressive disease, as in 33% of patients in our series. Even in 1 patient for whom CT imaging did not detect overt progression despite a rise in exoDNA *KRAS* MAF, laparotomy confirmed the discovery of CT-occult omental metastasis. These data suggest a role for serial liquid biopsies, and specifically exoDNA, as a putative predictive biomarker for disease status after neoadjuvant therapy.

It is important to note the following weaknesses of the current study. Although the strategy of using mutant *KRAS* molecules as a tumor marker may be theoretically optimal in a disease like PDAC, where *KRAS* mutation rates exceed 90%, the stochastic nature of circulating nucleic acids released in circulation may lead to underestimation of the true circulating tumor burden if detection is limited to a single mutation. This may likely be a contributing factor to the poor predictive potential of ctDNA in the context of metastatic disease and those patients receiving neoadjuvant therapy. We found notable differences in our previously published exo*KRAS* and ctDNA detection rates in patients with metastatic (85% and 57.9%, respectively) and localized disease (67% and 45.5%, respectively) obtained from a retrospective bio-banked cohort.[11] The differences in detection rates are largely due to the fact that exoDNA and ctDNA in the previous study underwent whole-genome amplification to increase sensitivity of *KRAS* detection in the context of early detection efforts. Although this was a possibility in the current series, we opted against amplification, because this would have distorted the MAFs found through ddPCR and thus affected our clinical endpoints. The use of a tumor gene panel (eg, *KRAS, TP53, CDKN2A,* and *SMAD4)* may achieve greater sensitivity for detection and monitoring.[2,6] Additionally, the fact that our multigene panel does not cover *KRAS* hotspot mutations in codon 61 may lead to underestimation of our true sensitivity, because the current panel has a theoretical detection rate of up to 80% of known *KRAS* mutations in PDAC.[30] Although our detection rates of *KRAS* mutant molecules are relatively modest at 32%−41% in baseline treatment-

Author Manuscript Author Manuscript Author Manuscript Author Manuscript

naïve metastatic patients based on the liquid biopsy compartment, a fact that may limit the number of patients who may benefit from such an assay, when we look at general detection in both compartments at once, detection rates increase to 73.1%, which is near the theoretical limit of our assay. This underlines the complementary nature of these biomarkers, especially in the setting of low-volume disease (such as after treatment or when monitoring for recurrence), whereby the absence of mutant detection in one does not preclude the ability to gain valuable genomic information in the other. Additionally, although exoDNA mutant *KRAS* detection levels compared with ctDNA detection levels are not significantly better in the current cohort, exosomes provide the added ability to perform specific enrichment of cancer-derived material, allowing for capture of DNA, RNA, and proteins derived from tumors for mutation, gene expression, and possibly even neoantigen detection.[14] The need for a criterion standard validation is also important when pursuing liquid biopsy assays such as the one described in this study. As such, recent work has attempted to validate concordance between mutations found in liquid biopsy and tissue biopsy samples.[31–36] In the context of PDAC, acquiring tissue biopsy samples for molecular profiling is particularly difficult in the metastatic setting, where fine needle aspirates are typically reserved for diagnostic purposes. We thus selected a subset of 34 patients with localized disease with available matched tissues, where concordance rates ranged from 66.7% to 95.5% depending on the liquid biopsy platform and tissue source. Unsurprisingly, surgical tissue specimens showed greater rates of concordance, particularly in exoDNA, which is likely associated with the greater sensitivity of mutation detection in exosomes. Overall, KRAS mutation detection rates were high in liquid biopsy samples as a whole, although it remains to be seen if profiling of additional mutations can achieve this sensitivity and specificity.

In conclusion, our study in a relatively large cohort of PDAC patients, composed of both metastatic and localized disease, reiterates the predictive and prognostic value of liquid biopsies in this malignancy. We show that although the baseline CA19–9, exoDNA, and ctDNA cargo has prognostic effect, longitudinal monitoring of exoDNA provides unique predictive information on the outcome of neoadjuvant therapy in localized disease and in anticipating progression in the metastatic setting. In contrast to the challenges of repetitive tissue biopsies for visceral cancers, serial liquid biopsies may provide an attractive alternative strategy to map tumor evolution in real time, providing an unprecedented insight into how the PDAC genome adapts to and eventually becomes recalcitrant to therapy.

Supplementary Material

Refer to Web version on PubMed Central for supplementary material.

Acknowledgments

Author contributions: Hector A. Alvarez and Anirban Maitra contributed to the study concept and design; Vincent Bernard, Dong U. Kim, Jonathan Castillo, Kelvin Allenson, Feven C. Mulu, Jun Zhao, Mark W. Hurd, and Bret M. Stephens contributed to acquisition of data; Vincent Bernard, Dong U. Kim, F. Anthony San Lucas, Joseph M. Herman, Alexander Semaan, Paola A. Guerrero, Nabiollah Kamyabi, Matthew Katz, Eugene J. Koay, Cullen M. Taniguchi, Milind Javle, Gauri Varadhachary, Anirban Maitra, and Hector A. Alvarez contributed to analysis and interpretation of data; Vincent Bernard, Dong U. Kim, and F. Anthony San Lucas drafted the manuscript; Vincent Bernard, Dong U. Kim, F. Anthony San Lucas, Jonathan Castillo, Joseph M. Herman, Alexander Semaan, Paola A. Guerrero, N.B., Matthew Katz, Eugene J. Koay, Cullen M. Taniguchi, Milind Javle, Gauri Varadhachary, Anirban Maitra, and Hector A. Alvarez performed critical revision of the manuscript for important intellectual content;

statistical analysis was done by Vincent Bernard, F. Anthony San Lucas, Dong U. Kim, and Joseph M. Herman; Anirban Maitra obtained funding support; Mark W. Hurd and Jun Zhao provided administrative support; and study supervision was supported by Katz, Mark W. Hurd, Eugene J. Koay, Cullen M. Taniguchi, Milind Javle, Gauri Varadhachary, Anirban Maitra, and Hector A. Alvarez.

Funding

This work was supported by the MD Anderson Moonshot Program and the Khalifa Bin Zayed Al-Nahyan Foundation (no grant numbers apply); the National Institutes of Health (U01CA196403 and U01CA200468 to Anirban Maitra); the Cancer Prevention Research Institute of Texas (RP160517 to Anirban Maitra; Vincent Bernard and Nabiollah Kamyabi were funded through a fellowship from Cancer Prevention Research Institute of Texas Research Training Program RP170067; and the German Research Foundation (SE- 2616/2-1 to Alexander Semaan). We are grateful the Precision Medicine Research Associates/Fox Family Foundation for partially supporting the studies described in this manuscript.

Abbreviations used in this paper:

**CA**	carbohydrate antigen
**CI**	confidence interval
**CT**	computed tomographic
**ctDNA**	circulating tumor DNA
**ddPCR**	digital droplet polymerase chain reaction
**exoDNA**	exosome-derived DNA
**MAF**	mutant allelic fraction
**OR**	odds ratio
**OS**	overall survival
**PBS**	phosphate-buffered saline
**PDAC**	pancreatic ductal adenocarcinoma
**PFS**	progression-free survival

References

1. Rahib L, Smith BD, Aizenberg R, et al. Projecting cancer incidence and deaths to 2030: the unexpected burden of thyroid, liver, and pancreas cancers in the United States. Cancer Res 2014;74:2913–2921. [PubMed: 24840647]
2. Chaudhuri AA, Chabon JJ, Lovejoy AF, et al. Early detection of molecular residual disease in localized lung cancer by circulating tumor DNA profiling. Cancer Discov 2017;9(12):CD-17–0716.
3. Beaver JA, Jelovac D, Balukrishna S, et al. Detection of cancer DNA in plasma of patients with early-stage breast cancer. Clin Cancer Res 2014;20:2643–2650. [PubMed: 24504125]
4. Garcia-Murillas I, Schiavon G, Weigelt B, et al. Mutation tracking in circulating tumor DNA predicts relapse in early breast cancer. Sci Transl Med 2015;7:302ra133.
5. Tie J, Wang Y, Tomasetti C, et al. Circulating tumor DNA analysis detects minimal residual disease and predicts recurrence in patients with stage II colon cancer. Sci Transl Med 2016;8:346ra92.
6. Abbosh C, Birkbak NJ, Wilson GA, et al. Phylogenetic ctDNA analysis depicts early-stage lung cancer evolution. Nature 2017;545:446–451. [PubMed: 28445469]

Author Manuscript Author Manuscript Author Manuscript Author Manuscript

Author Manuscript Author Manuscript Author Manuscript Author Manuscript

7. Bettegowda C, Sausen M, Leary RJ, et al. Detection of circulating tumor DNA in early- and late-stage human malignancies. Sci Transl Med 2014;6:224ra24.
8. Le Calvez-Kelm F, Foll M, Wozniak MB, et al. KRAS mutations in blood circulating cell-free DNA: a pancreatic cancer case-control. Oncotarget 2016;7:78827–78840. [PubMed: 27705932]
9. Pietrasz D, Pecuchet N, Garlan F, et al. Plasma circulating tumor DNA in pancreatic cancer patients is a prognostic marker. Clin Cancer Res 2017;23:116–123. [PubMed: 27993964]
10. Takai E, Totoki Y, Nakamura H, et al. Clinical utility of circulating tumor DNA for molecular assessment and precision medicine in pancreatic cancer. Adv Exp Med Biol 2016;924:13–17. [PubMed: 27753011]
11. Allenson K, Castillo J, San Lucas FA, et al. High prevalence of mutant KRAS in circulating exosome-derived DNA from early-stage pancreatic cancer patients. Ann Oncol 2017;28:741–747. [PubMed: 28104621]
12. San Lucas FA, Allenson K, Bernard V, et al. Minimally invasive genomic and transcriptomic profiling of visceral cancers by next-generation sequencing of circulating exosomes. Ann Oncol 2016;27:635–641. [PubMed: 26681674]
13. Kahlert C, Melo SA, Protopopov A, et al. Identification of double-stranded genomic DNA spanning all chromosomes with mutated KRAS and p53 DNA in the serum exosomes of patients with pancreatic cancer. J Biol Chem 2014;289:3869–3875. [PubMed: 24398677]
14. Castillo J, et al. Surfaceome profiling enables isolation of cancer-specific exosomal cargo in liquid biopsies from pancreatic cancer patients. Ann Oncol 2018;29: 223–229. [PubMed: 29045505]
15. Chun YS, Pawlik TM, Vauthey JN. 8th Edition of the AJCC Cancer Staging Manual: Pancreas and Hepatobiliary Cancers. Ann Surg Oncol 2018;25:845–847. [PubMed: 28752469]
16. Eisenhauer EA, Therasse P, Bogaerts J, et al. New response evaluation criteria in solid tumours: revised RECIST guideline (version 1.1). Eur J Cancer 2009;45:228–247. [PubMed: 19097774]
17. Thakur BK, Zhang H, Becker A, et al. Double-stranded DNA in exosomes: a novel biomarker in cancer detection. Cell Res 2014;24:766–769. [PubMed: 24710597]
18. Furukawa T, Kuboki Y, Tanji E, et al. Whole-exome sequencing uncovers frequent GNAS mutations in intraductal papillary mucinous neoplasms of the pancreas. Sci Rep 2011;1:161. [PubMed: 22355676]
19. Lee JH, Kim Y, Choi JW, et al. KRAS, GNAS, and RNF43 mutations in intraductal papillary mucinous neoplasm of the pancreas: a meta-analysis. Springerplus 2016; 5:1172. [PubMed: 27512631]
20. Luttges J, Diederichs A, Menke MA, et al. Ductal lesions in patients with chronic pancreatitis show K-ras mutations in a frequency similar to that in the normal pancreas and lack nuclear immunoreactivity for p53. Cancer 2000; 88:2495–2504. [PubMed: 10861425]
21. Maire F, Micard S, Hammel P, et al. Differential diagnosis between chronic pancreatitis and pancreatic cancer: value of the detection of KRAS2 mutations in circulating DNA. Br J Cancer 2002;87:551–554. [PubMed: 12189555]
22. Mohrmann L, Huang H, Hong DS, et al. Liquid biopsies using plasma exosomal nucleic acids and plasma cell- free DNA compared with clinical outcomes of patients with advanced cancers. Clin Cancer Res 2017;24: CCR-17–2007.
23. Earl J, Garcia-Nieto S, Martinez-Avila JC, et al. Circulating tumor cells (Ctc) and kras mutant circulating free DNA (cfDNA) detection in peripheral blood as biomarkers in patients diagnosed with exocrine pancreatic cancer. BMC Cancer 2015;15:797. [PubMed: 26498594]
24. Hadano N, Murakami Y, Uemura K, et al. Prognostic value of circulating tumour DNA in patients undergoing curative resection for pancreatic cancer. Br J Cancer 2016;115:59–65. [PubMed: 27280632]
25. Cohen JD, Javed AA, Thoburn C, et al. Combined circulating tumor DNA and protein biomarker-based liquid biopsy for the earlier detection of pancreatic cancers. Proc Natl Acad Sci U S A 2017;114:10202–10207. [PubMed: 28874546]
26. Cohen JD, Li L, Wang Y, et al. Detection and localization of surgically resectable cancers with a multi-analyte blood test. Science 2018;359:926–930. [PubMed: 29348365]

Author Manuscript Author Manuscript Author Manuscript Author Manuscript

27. Tjensvoll K, Lapin M, Buhl T, et al. Clinical relevance of circulating KRAS mutated DNA in plasma from patients with advanced pancreatic cancer. Mol Oncol 2016; 10:635–643. [PubMed: 26725968]

28. Sausen M, Phallen J, Adleff V, et al. Clinical implications of genomic alterations in the tumour and circulation of pancreatic cancer patients. Nat Commun 2015;6:7686. [PubMed: 26154128]

29. Cloyd JM, Wang H, Egger ME, et al. Association of clinical factors with a major pathologic response following preoperative therapy for pancreatic ductal adenocarcinoma. JAMA Surg 2017;152:1048–1056. [PubMed: 28700784]

30. Cerami E, Gao J, Dogrusoz U, et al. The cBio cancer genomics portal: an open platform for exploring multidimensional cancer genomics data. Cancer Discov 2012; 2:401–404. [PubMed: 22588877]

31. Chae YK, Davis AA, Jain S, et al. Concordance of genomic alterations by next-generation sequencing in tumor tissue versus circulating tumor DNA in breast cancer. Mol Cancer Ther 2017;16:1412–1420. [PubMed: 28446639]

32. Schwaederle M, Husain H, Fanta PT, et al. Use of liquid biopsies in clinical oncology: pilot experience in 168 patients. Clin Cancer Res 2016;22:5497–5505. [PubMed: 27185373]

33. Villaflor V, Won B, Nagy R, et al. Biopsy-free circulating tumor DNA assay identifies actionable mutations in lung cancer. Oncotarget 2016;7:66880–66891. [PubMed: 27602770]

34. Hahn AW, Gill DM, Maughan B, et al. Correlation of genomic alterations assessed by next-generation sequencing (NGS) of tumor tissue DNA and circulating tumor DNA (ctDNA) in metastatic renal cell carcinoma (mRCC): potential clinical implications. Oncotarget 2017;8:33614–33620. [PubMed: 28431395]

35. Kuderer NM, Burton KA, Blau S, et al. Comparison of 2 commercially available next-generation sequencing platforms in oncology. JAMA Oncol 2017;3:996–998. [PubMed: 27978570]

36. Toor OM, Ahmed Z, Bahaj W, et al. Correlation of somatic genomic alterations between tissue genomics and ctDNA employing next generation sequencing: analysis of lung and gastrointestinal cancers. Mol Cancer Ther 2018;144:2167–2175.

Author Manuscript Author Manuscript Author Manuscript Author Manuscript

WHAT YOU NEED TO KNOW

BACKGROUND AND CONTEXT

There is a crucial need for effective strategies for real-time monitoring during pancreatic cancer treatment, to identify chemorefractory status and direct therapy.

NEW FINDINGS

Serial quantitative measurements of circulating nucleic acid sources can provide clinically relevant predictive and prognostic information in pancreatic cancer patients, including anticipation of impending disease progression.

LIMITATIONS

Future, prospective studies using larger patient cohorts are needed to validate the metrics used for therapy progression and their clinical relevance.

IMPACT

"Liquid biopsies" using circulating DNA may provide a minimally invasive strategy for informing therapeutic decision-making in pancreatic cancer.

Author Manuscript Author Manuscript Author Manuscript Author Manuscript

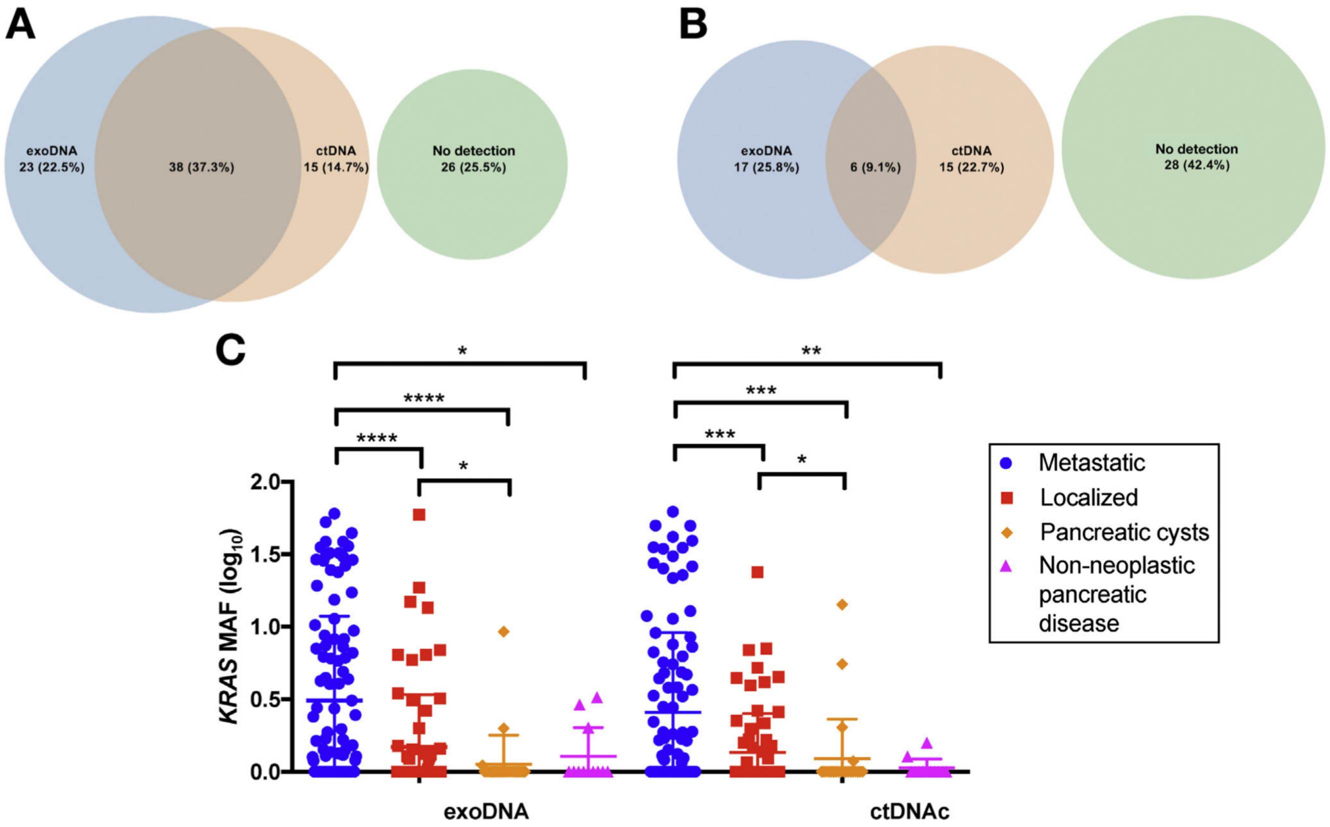

Figure 1.

Venn diagram of detection rates of codon 12/13 mutant *KRAS* by ddPCR among (*A*) 102 patients with metastatic disease and (*B*) 66 patients with localized PDAC with matched exoDNA and ctDNA analysis. (*C*) MaF of *KRAS* mutations detected through ddPCR in exoDNA and ctDNA among baseline treatment-naïve patients with localized and metastatic disease and patients with pancreatic cysts and nonneoplastic pancreatic disease. Greater median MAF in exoDNA compared with ctDNA in patients with metastatic disease trended toward significance ($P = .05$); paired analysis was performed by Wilcoxon test. Those patients with metastatic disease had higher *KRAS* MAF in both exoDNA ($P < .0001$) and ctDNA (P = .0004) than patients with localized disease, by Mann–Whitney test. *$P < .05$, **$P < .01$, ***$P < .001$, ****$P < .0001$.

Author Manuscript
Author Manuscript
Author Manuscript
Author Manuscript

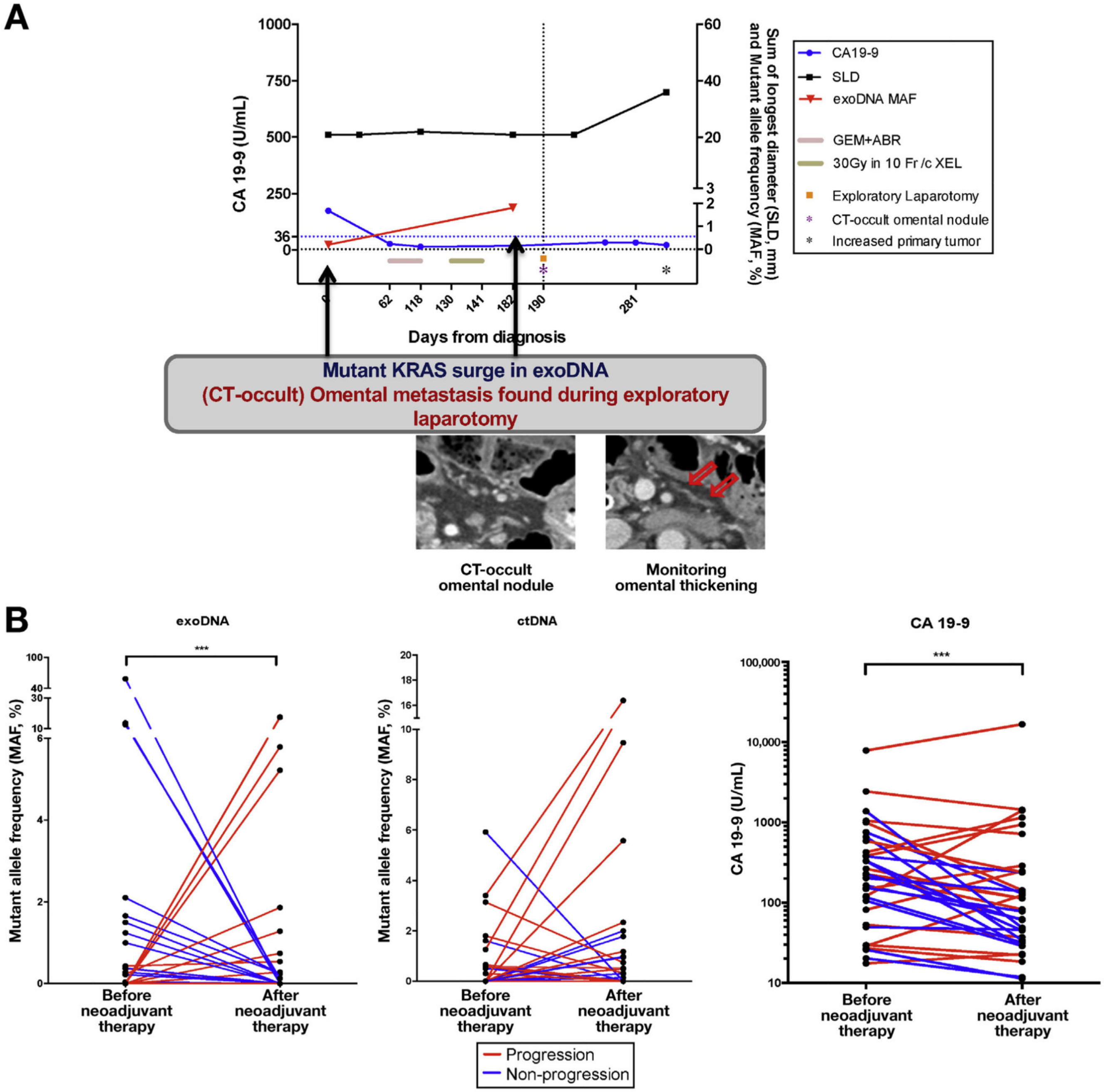

Figure 2.

(*A*) Tumor monitoring before and after neoadjuvant chemoradiation in a patient experiencing progression undetectable by CA19–9 (blue line) or radiologic-based RECIST 1.1 (black line). (*B*) MAF kinetics of exoDNA and ctDNA before and after neoadjuvant therapy show a significant correlation between a rise or no change in exoDNA MAF and progression (OR, 38.4; 95% Ci, 3.95–373.3; $P = .0002$). No significant correlation was detectable by ctDNA. Changes in CA19–9 from baseline were also significantly associated to progressive disease (OR, 28.0; 95% CI, 2.65–295.9; $P = .003$). ABR, abraxane; GEM, gemcitabine; SLD, sum of longest diameters.

Author Manuscript

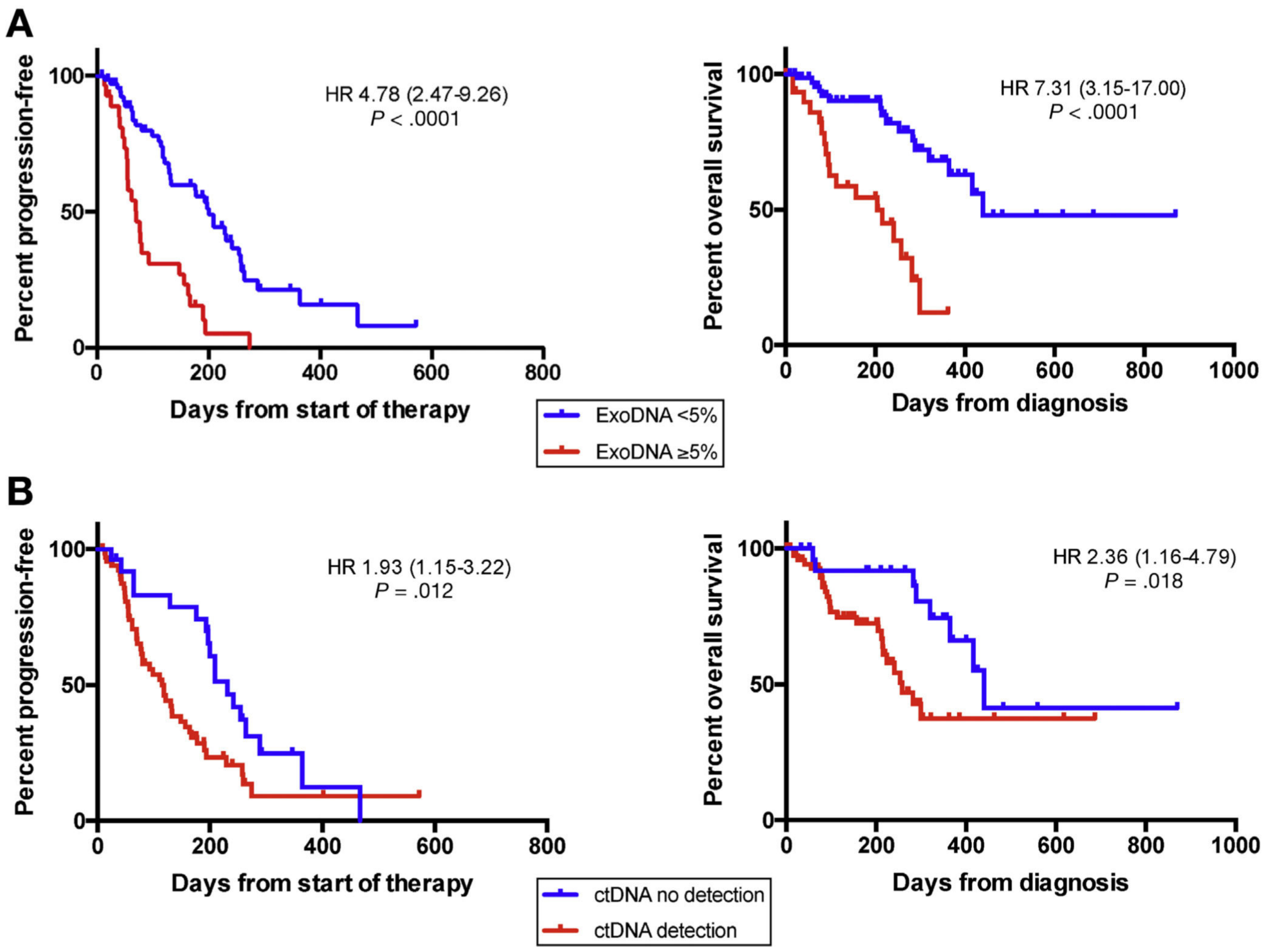

Figure 3.

Kaplan-Meier curve stratification of baseline treatment-naïve patients based on tumor burden as measured by exoDNA. (*A*) Patients with exo*KRAS* ≥5% experienced worse PFS (median, 71 vs 200 days) and OS (median, 204 vs 440 days). Detection of ctDNA was significantly associated with worse PFS (median, 118 vs 231 days) and OS (median, 258 days vs 440 days).

Author Manuscript

Author Manuscript

Author Manuscript

Author Manuscript

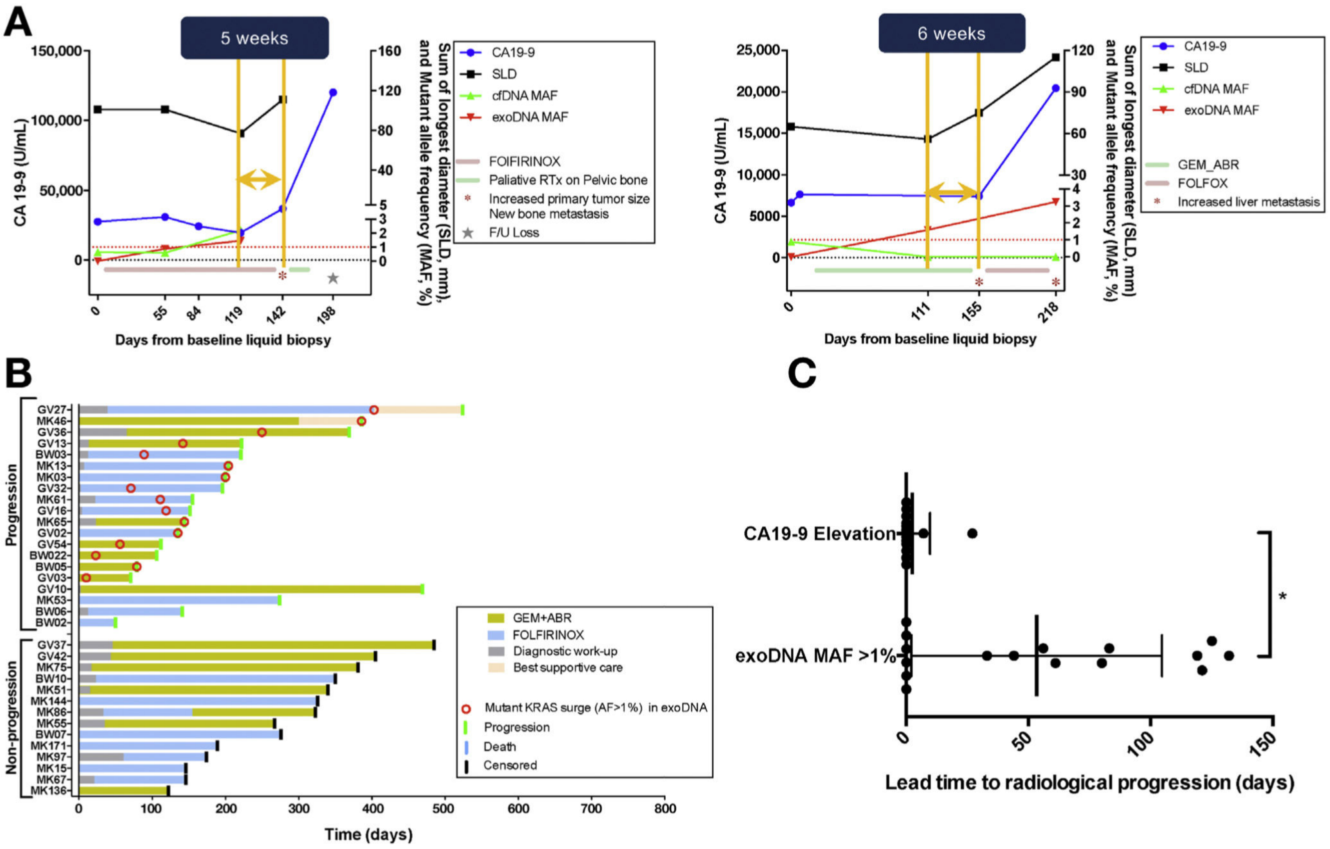

Figure 4.

(*A*) Tumor monitoring using serial liquid biopsies showing correlation between a exo*KRAS* peak ≥1% (red line) and radiologic progression based on RECIST 1.1 (black line). The standard pancreatic cancer biomarker is plotted (blue), as is ct*KRAS* (green) for comparison. (*B*) Tumor monitoring among 34 patients shows the ability of exo*KRAS* peaks ≥1% (red circle) to predict radiologic progression (green bar). Peaks are identified in 11 of 14 patients who progressed vs in no patients who did not progress (9/9). exo*KRAS* peak is significantly associated with progression (P = .0003) on Fisher exact test with an odds ratio of 62.4 (95% CI, 2.852–1367). (*C*) MAF *KRAS* peak shows a significantly greater median lead time in predicting progression of 50 days (P = .03) from the time clinically detectable progression was evident on CT imaging compared with CA19– 9 (median lead time, 0 days). ABR, abraxane; AF, allelic fraction; FOLFOX, folinic acid, fluorouracil, oxaliplatin; GEM, gemcitabine, SLD, sum of longest diameters.
