# Supplementary material for: Longitudinal monitoring of KRAS-mutated circulating tumor DNA enables the prediction of prognosis and therapeutic responses in patients with pancreatic cancer
Source: PLoS One. 2019 Dec 31;14(12):e0227366. doi: 10.1371/journal.pone.0227366 (PMC6938323; doi:10.1371/journal.pone.0227366)
Supplement: Attachment — Submitted filename: paper review Plos One.docx [file pone.0227366.s009.docx]

**General comments:**

The article entitled **“longitudinal monitoring of KRAS-mutated circulating tumor DNA enables the prediction of prognosis and therapeutic responses in patients with pancreatic cancer”** describes the significance of mutated KRAS ctDNA as a prognostic marker in the liquid biopsy samples of pancreatic cancer patients. I appreciate the efforts from the authors for performing the in-depth analysis. Because the similar work has been published previously with some of the closely matching conclusions, the submitted manuscript need to be redrafted before considering it for publication. In addition, there are major concerns related to manuscript writing, reference citation, and plagiarism. If considered for publication, following are my comments that need to be addressed:

**Major comments:**

1. Although the study has been performed on a bigger cohort of PC patients and include the longitudinal analysis of liquid biopsy samples, authors need to justify how the submitted manuscript is different than the previously published research on prognostic significance of mutKRAS ctDNA in PC patients. following are the references for similar kind of work:

- Kruger, S., et al., *Repeated mutKRAS ctDNA measurements represent a novel and promising tool for early response prediction and therapy monitoring in advanced pancreatic cancer.* Ann Oncol, 2018. **29**(12): p. 2348-2355.
- Cohen, J.D., et al., *Combined circulating tumor DNA and protein biomarker-based liquid biopsy for the earlier detection of pancreatic cancers.* Proceedings of the National Academy of Sciences, 2017. **114**(38): p. 10202.
- Tjensvoll, K., et al., *Clinical relevance of circulating KRAS mutated DNA in plasma from patients with advanced pancreatic cancer.* Molecular Oncology, 2016. **10**(4): p. 635-643.

2. The abstract has been previously published in JCO and content has been copied in the submitted manuscript. It will be considered as self-plagiarism and therefore, authors are requested to modify the abstract [*DOI: 10.1200/JCO.2019.37.15_suppl.e15712 Journal of Clinical Oncology 37, no. 15_suppl*].
3. Authors focused on comparative analysis of mutKRAS ctDNA and CA19-9 in liquid biopsy samples ignoring their combined sensitivity in prediction of prognosis. Previous study by Cohen et al., 2017 in PNAS states that combination of ctDNA and protein-based markers increases the sensitivity.
4. The references are neither updated nor cited appropriately, e.g. ref 01 is about pancreatic cancer statistics and it was published in 2009, although 2019 cancer statistics is available.
5. The manuscript needs scientific writing with no repetition of sentences to maintain proper connectivity and flow of information.
6. Citations in the manuscript do not look appropriate as all the citations are placed after the period, which need to be corrected.

**Specific comments:**

1. Line 24-25; modify as “All of the other authors contributed to sample collection, data collection and interpretation, and manuscript review”.
2. Line 240 and 356; patients without……….need to be elaborated.
3. Line 255-257; Highlighted in bold and sentence finished abruptly………….correction required.
4. All figure legends are inserted in the main text. Please submit the separate file for figure legends.
5. Line 364-365; remove the bold font and put a period at the end of the sentence.
6. Pancreatic cancer has been used multiple time and it could be abbreviated as PC.
7. Line 412-415; sentence has been repeated, correction needed.
8. Line 441-444; sentence has been repeated, correction needed.
9. There are relevant and recent references are missed by the authors therefore, references need to be updated.
10. Figure 2; better resolution is required so that in print form, it should be readable.
11. Supporting information; sentences are not ending with the period, written casually. Please revise the supporting information.
12. Figure legends: Figure legends are not correctly written. X-and Y-axes are not defined in the legends and there is no statistical information mentioned in the legends.
13. For data reproducibility, authors have not mentioned how many times the experiments were repeated to get the statistical significance. While writing the figure legends, please mention how many times the experiment were repeated with their statistical significance.
