# Supplementary material for: Longitudinal monitoring of KRAS-mutated circulating tumor DNA enables the prediction of prognosis and therapeutic responses in patients with pancreatic cancer
Source: PLoS One. 2019 Dec 31;14(12):e0227366. doi: 10.1371/journal.pone.0227366 (PMC6938323; doi:10.1371/journal.pone.0227366)
Supplement: Attachment — Submitted filename: Response_to_Reviewers_Watanabe_L5.docx [file pone.0227366.s011.docx]

November 15, 2019

PONE-D-19-17596
Title: Longitudinal monitoring of *KRAS*-mutated circulating tumor DNA enables the prediction of prognosis and therapeutic responses in patients with pancreatic cancer

Dear Professor Joerg Heber,

We express our gratitude for your letter dated August 22, 2019, along with the detailed and constructive comments from reviewers on our submitted manuscript titled “Longitudinal monitoring of *KRAS*-mutated circulating tumor DNA enables the prediction of prognosis and therapeutic responses in patients with pancreatic cancer.”

We thank the reviewers for their thoughtful suggestions and insights. The manuscript has benefited from these insightful suggestions and has much improved after incorporation of the reviewers’ comments. We have amended the manuscript in accordance with the reviewers’ suggestions. My point-by-point responses to all reviewers’ comments have been prepared and provided in the subsequent pages of this letter, saved as “Response to Reviewers” file. The reviewers’ original comments are indicated as blue text, whereas my responses are shown in black, with each modified point displayed as red underlined text. A marked-up copy of the changes made from the previous manuscript file has been saved as “Revised Manuscript with Track Changes” file. A clean revised manuscript has been saved as “Manuscript” file. We would prefer to remove my manuscript from collection consideration.

The present study was supported by Grant-in-Aid for Scientific Research (grant number JP 16K10514) from the Ministry of Education, Culture, Sports, Science and Technology and the JKA Foundation through its promotion funds from the Keirin Race (grant number 27-1-068 (2)).

The funders had no role in study design, data collection and analysis, decision to publish, or preparation of the manuscript

The authors have declared that no competing interests exist.

The authors declare that we have no significant competing financial, professional, or personal interests that might have influenced the performance or presentation of the work described in this manuscript.

Sincerely yours,

Koichi Suzuki, M.D.

Department of Surgery, Saitama Medical Center

Jichi Medical University

1-847, Amanuma-cho, Omiya-ku, Saitama 330-8503, Japan

Tel.: 81-48-647-2111

Fax: 81-48-644-8617

E-mail: [ksuzbnhm@yahoo.co.jp](mailto:ksuzbnhm@yahoo.co.jp)

**Response to PLOS Editorial Manager**

1. In the Methods, please provide a justification of the sample size used in this study.

In accordance with the editor’s suggestion, we have revised the manuscript as follows:

Methods section, lines 130–131

This study is conducted as an exploratory study without calculation of sample size for primary endpoints.

2. Our staff editors have determined that your manuscript is likely within the scope of our Targeted Anticancer Therapies and Precision Medicine Call for Papers. This editorial initiative is headed by a team of Guest Editors for PLOS ONE: Andrew Cherniack, Anette Duensing, Steven Gray, Sunil Krishnan, Chandan Kumar-Sinha and Gayle Woloschak. The Collection will encompass a diverse range of research articles about the identification and classification of driver genes and somatic alterations, target and drug discovery, mechanisms of drug resistance, and early detection and screening. Additional information can be found on our announcement page: <https://collections.plos.org/s/targeted-anticancer-therapies>.

We would prefer to remove my manuscript from collection consideration.

3. Thank you stating the following in your competing interests statement:

"“The funders had no role in study design, data collection and analysis, decision to publish, or preparation of the manuscript”"

a. Please provide a full competing interests statement. If NO authors have competing interests, please state: "The authors have declared that no competing interests exist."

"The authors have declared that no competing interests exist."

If Authors have competing interests please enter competing interest details beginning with this statement: "I have read the journal's policy and the authors of this manuscript have the following competing interests: [insert competing interests here]"

b. Please include your updated Competing Interests statement in your cover letter; we will change the online submission form on your behalf.

The authors declare that we have no significant competing financial, professional, or personal interests that might have influenced the performance or presentation of the work described in this manuscript.

**Responses to Reviewers’ Comments**

**General comments:**

The article entitled **“longitudinal monitoring of KRAS-mutated circulating tumor DNA enables the prediction of prognosis and therapeutic responses in patients with pancreatic cancer”** describes the significance of mutated KRAS ctDNA as a prognostic marker in the liquid biopsy samples of pancreatic cancer patients. I appreciate the efforts from the authors for performing the in-depth analysis. Because the similar work has been published previously with some of the closely matching conclusions, the submitted manuscript need to be redrafted before considering it for publication. In addition, there are major concerns related to manuscript writing, reference citation, and plagiarism. If considered for publication, following are my comments that need to be addressed:

**Major comments:**

1. Although the study has been performed on a bigger cohort of PC patients and include the longitudinal analysis of liquid biopsy samples, authors need to justify how the submitted manuscript is different than the previously published research on prognostic significance of mutKRAS ctDNA in PC patients. following are the references for similar kind of work:

- Kruger, S., et al., *Repeated mutKRAS ctDNA measurements represent a novel and promising tool for early response prediction and therapy monitoring in advanced pancreatic cancer.* Ann Oncol, 2018. **29**(12): p. 2348-2355.

We are in agreement the editor’s suggestion. Kruger et al. reported that a decrease in ^mut^*KRAS* ctDNA levels during therapy was an early indicator of response to therapy during the first 4 weeks of treatment. In accordance with the reviewer’s suggestion, we have revised the manuscript as follows:

Discussion section, lines 404-406

“These results are congruent with those reported by Del Re et al. [25] and Kruger et al. [43] who showed that early changes in *KRAS-*mutated ctDNA levels were useful for monitoring treatment responses in patients with PDAC.”

- Cohen, J.D., et al., *Combined circulating tumor DNA and protein biomarker-based liquid biopsy for the earlier detection of pancreatic cancers.* Proceedings of the National Academy of Sciences, 2017. **114**(38): p. 10202.

Cohen et al. aimed to detect the early stage of pancreatic cancer by combined circulating tumor DNA and protein biomarker-based liquid biopsy. The aim of their study is slightly different from the purpose of our study; hence, this paper was not cited in our manuscript.

- Tjensvoll, K., et al., *Clinical relevance of circulating KRAS mutated DNA in plasma from patients with advanced pancreatic cancer.* Molecular Oncology, 2016. **10**(4): p. 635-643.

In the study by Tjensvoll et al., Kaplan–Meier survival analyses indicated that patients with positive ctDNA status before or after initiation of chemotherapy had shorter progression-free survival, albeit without statistical significance.

2. The abstract has been previously published in JCO and content has been copied in the submitted manuscript. It will be considered as self-plagiarism and therefore, authors are requested to modify the abstract [*DOI: 10.1200/JCO.2019.37.15_suppl.e15712 Journal of Clinical Oncology 37, no. 15_suppl*].

In view of your valuable comment, we have accordingly revised the abstract and modified it in a different form.

3. Authors focused on comparative analysis of mutKRAS ctDNA and CA19-9 in liquid biopsy samples ignoring their combined sensitivity in prediction of prognosis. Previous study by Cohen et al., 2017 in PNAS states that combination of ctDNA and protein-based markers increases the sensitivity.

Cohen et al. focused on detecting the early stage of pancreatic cancer by combined circulating tumor DNA and protein biomarker-based liquid biopsy. Our study aimed to evaluate the usefulness of *KRAS* ctDNA monitoring for the prediction of prognosis and therapeutic responses in patients with pancreatic cancer.

4. The references are neither updated nor cited appropriately, e.g. ref 01 is about pancreatic cancer statistics and it was published in 2009, although 2019 cancer statistics is available.

I apologize for the inappropriate reference. We have accordingly replaced the original reference with the reference #2 for the paper published in 2019.

5. The manuscript needs scientific writing with no repetition of sentences to maintain proper connectivity and flow of information.

I apologize for the repetition of some sentences. In view of your comment, we have accordingly revised the manuscript.

6. Citations in the manuscript do not look appropriate as all the citations are placed after the period, which need to be corrected.

I apologize for this. As per your advice, we have corrected this matter in the revised manuscript, and all reference citations are now placed before the period or comma.

**Specific comments:**

1. Line 24-25; modify as “All of the other authors contributed to sample collection, data collection and interpretation, and manuscript review”.

In accordance with the reviewer’s suggestion, we have revised the manuscript as follows:

Title page, lines 24–25

“All of the other authors contributed to sample collection, data collection and interpretation, and manuscript review.”

2. Line 240 and 356; patients without……….need to be elaborated.

Based on the reviewer’s suggestion, we have revised the manuscript as follows:

Results section, lines 239–240

“These 8 patients without *KRAS* mutation detected by RASKET showed the presence of *KRAS* mutations by ddPCR.”

Results section, lines 325

“hence, we re-evaluated the outcome by comparing patients with emergence of ctDNA within 1 year to patients without emergence.”

3. Line 255-257; Highlighted in bold and sentence finished abruptly………….correction required.

In accordance with the reviewer’s suggestion, we have revised the manuscript as follows:

Results section, lines 253–255:

“**One-point assessment of** ***KRAS* ctDNA and CA19-9 levels before surgery was not associated with RFS, but *KRAS* ctDNA before chemotherapy was a potential predictive prognostic factor, whereas CA19-9 prior to chemotherapy was** **not**”

4. All figure legends are inserted in the main text. Please submit the separate file for figure legends.

I apologize for the inappropriate insertion of figure legends in the main text. Figure legends have now been placed at the end of the manuscript.

5. Line 364-365; remove the bold font and put a period at the end of the sentence.

As per the reviewer’s suggestion, we have revised the relevant text in the manuscript as follows:

Results section, lines 329–330:

“**Sequential assessments of ctDNA within 6 months to assess therapeutic responses of chemotherapy-naïve patients**”

6. Pancreatic cancer has been used multiple time and it could be abbreviated as PC.

In view of the reviewer’s comment, we have replaced the term “pancreatic cancer” in the text with the abbreviation “PDAC.”

7. Line 412-415; sentence has been repeated, correction needed.

I apologize for the repeated sentence. We have accordingly deleted this in the manuscript as follows:

Discussion section, lines 373

“~~reported that one-point assessment of ctDNA before surgery was associated with patient outcome.~~”

8. Line 441-444; sentence has been repeated, correction needed.

We did not identify any repeated sentence in lines 441–444.

9. There are relevant and recent references are missed by the authors therefore, references need to be updated.

I apologize for the inappropriate references. In view of the reviewer’s comment, we have accordingly checked all references and appropriately updated them.

10. Figure 2; better resolution is required so that in print form, it should be readable.

In accordance with the reviewer’s advice, we have replaced the figure with Figure 2 with better resolution.

11. Supporting information; sentences are not ending with the period, written casually. Please revise the supporting information.

I apologize for this. We have accordingly revised the sentences in view of your comment.

12. Figure legends: Figure legends are not correctly written. X-and Y-axes are not defined in the legends and there is no statistical information mentioned in the legends.

In accordance with the reviewer’s comment, we have revised the figure legends and defined the X- and Y-axes.

13. For data reproducibility, authors have not mentioned how many times the experiments were repeated to get the statistical significance. While writing the figure legends, please mention how many times the experiment were repeated with their statistical significance.

In view of the reviewer’s comment, we have added the following text in the Methods section:

Lines 194–195:

“For data reproducibility, analysis of *KRAS* status in tumor tissues and plasma was performed in duplicate or triplicate.”
